# Live Visualization of Endoplasmic Reticulum Redox Potential in Zebrafish Embryos Reveals Region-Specific Heterogeneity

**DOI:** 10.3390/biomedicines14071585

**Published:** 2026-07-15

**Authors:** Monika Verma, Niraj Rajesh Bhatt, Koushika Chandrasekaran, Aseem Chaphalkar, Kriti Verma, Shreyansh Umale, Shweta Verma, Chetana Sachidanandan, Kausik Chakraborty

**Affiliations:** 1 CSIR-Institute of Genomics and Integrative Biology, Mathura Road, New Delhi 110025, Indiaaseem.chaphalkar@gmail.com (A.C.);; 2 Academy of Scientific and Innovative Research (AcSIR), Coordination Office, CSIR-Human Resource Development Centre Campus, Ghaziabad 201002, UP, India; 3 Nuclear Dynamics and Cancer Program, Fox Chase Cancer Center, 333 Cottman Avenue, Philadelphia, PA 19111, USA; 4 Department of Biotechnology, Indian Institute of Technology Madras, Chennai 600036, TN, India

**Keywords:** redox homeostasis, roGFP, redox potential, signal sequence, zebrafish, reducing ER, oxidizing cytosol

## Abstract

**Objective:** Redox homeostasis is an integral part of many cellular processes, and its perturbation is associated with conditions such as diabetes, aging, and neurodegenerative disorders. Redox homeostasis or redox potential in organelles is maintained within a particular range to facilitate the organelle-specific cellular redox reactions. Previous studies using yeast, cell systems, and nematodes have demonstrated that the Endoplasmic Reticulum (ER) has a more oxidizing environment, while the cytosol exhibits a reducing redox potential. However, we know very little about how universal this phenomenon is. **Methods:** We created transgenic zebrafish (*Danio rerio*) lines with roGFP sensors targeted to the ER and cytosol for studying physiological redox potential at the systems level. In the process, we also characterized the ER-targeting signal sequence in *D. rerio* for the first time. **Results:** Measurements of the redox state in live embryos found that the endoplasmic reticulum exhibits consistent deviations from its expected oxidizing redox state in multiple regions of the developing embryos. The ER is far more reduced than expected in certain tissues of the embryo, including certain regions of the brain. We confirmed this heterogeneity using another transgenic line expressing ER-targeted roGFPiE, a redox-sensitive GFP better suited to measuring changes in ER redox potential. We also observed the resilient nature of the ER redox state following tunicamycin (Tm) and Azetidine-2-carboxylic acid-induced proteostasis perturbations and only mild changes with Tm. **Conclusions:** While our study provides a first glimpse of the diversity in ER redox homeostasis, these unanticipated redox states of the ER will require new biological definitions.

## 1. Introduction

The endoplasmic reticulum (ER) is the first stop for the proteins going through the secretory pathway and it acts as a hub for oxidative protein folding in the cell [1]. Oxidative protein folding in the ER is mediated by redox buffers and oxidoreductases such as the Protein Disulfide Isomerases (PDIs) and ER Oxidoreductin 1 (ERO1), aided by the oxidizing redox potential in the ER [2]. While the ER is oxidizing, other compartments like the cytosol and the mitochondrial matrix maintain a reducing environment to prevent the resident enzymes from oxidation and to facilitate metabolic reactions [3,4,5,6]. Differences in the redox potential of the ER and cytosol can also be attributed to differences in ratios of reduced and oxidized glutathione (GSH:GSSG). The cytosol has many-fold higher concentrations of GSH:GSSG (10:1 to 100:1) as compared to the ER (3:1 or 1:1) [5,7,8]. However, maintaining redox potential is a complex process, and apart from enzymes, it also depends on the redox buffers present in the cell. The generation of live probes for redox sensing such as the roGFP variants have revolutionized the field. These sensors, targeted to various cellular organelles, have been used to study intracellular differences in redox potential in a number of model organisms such as *Saccharomyces cerevisiae*, *Caenorhabditis elegans*, mammalian cell lines, and *Mus musculus* [9,10,11,12,13,14,15].

The ER is multi-functional; in addition to protein folding, it also serves as a compartment for calcium storage and lipid biosynthesis [16,17,18,19]. Within a cell, the ER maintains distinct sub-regions for these functions [20,21]. Additionally, the ERs in different organs in the body have different demands on them [22]; e.g., the pancreatic cells are secretory in nature with nearly 1/3rd–1/5th of the proteome passing through the ER, making protein folding the major activity [23], whereas the ER in muscles serves more as a calcium storage hub [24,25,26]. Given this, we speculated that the ER in different tissues would adapt to the differences in demand and would exhibit differences in redox potential.

Studies in the fruit fly larvae (*Drosophila melanogaster*) and nematodes (*C. elegans*) have shown differences in cytosolic redox potential across different regions of the animal [27]. Apfeld and colleagues showed that insulin signaling was important for the regulation of redox potential in the cytosol of *C. elegans* [12]. Morimoto and group demonstrated that the ER becomes less oxidizing while the cytosol becomes less reducing in aging worms [28]. Most of these studies on redox potential, performed in invertebrate models, have focused on perturbations due to chemical or environmental cues, with the assumption that the ER redox potential is constant across tissue types [28]. Recent studies have focused on redox changes in various organelles, e.g., in periplasm (*Escherichia coli*) [29]; cytosol and mitochondria during cell division and death [30,31]; cytosol, mitochondria, and nucleus during cell differentiation [32]; and mitochondria due to myocardial infarction [33]. Zebrafish roGFP and HyPer7 reporters have been used to study redox events in the mitochondria, cytosol and nucleus during axonal injury [34], fin wounding [35], in cardiomyocytes [36], and in ontogeny [37]. However, we know very little about the physiological redox states of endoplasmic reticulum in the different cell types in a zebrafish embryo.

We chose zebrafish (*Danio rerio*) as the vertebrate model to explore the redox potential dynamics of the ER and cytosol, because the transparency of the embryos allows us to monitor redox states in the live animal using sensors such as roGFP. The embryonic anatomy and molecular pathways are well conserved from zebrafish to human. The chemical permeability of the embryos and larvae also makes it easy to expose them to small molecule perturbation. In this study, we generated transgenic zebrafish lines ubiquitously expressing the redox sensor, roGFP, targeted to the endoplasmic reticulum (ER) and cytosol. During this process, we also characterized the ER-targeting signal sequence in zebrafish for the first time. Overall, the ER and cytosol in most tissues had the expected redox states. However, there were interesting deviations from the norm in the brain and some other tissues. These differences in redox states became even more evident when we used roGFPiE, a more sensitive variant of roGFP. Thus, our organelle-targeted redox sensors allowed us to visualize and quantify the redox states of cytosol and ER in various tissues in a live vertebrate model. These tools we have generated would be valuable in exploring the mechanisms for maintaining different redox states in different tissues and would also allow us to understand the changes in redox states induced under pathophysiological conditions.

## 2. Materials and Methods

### 2.1. Zebrafish Maintenance and Breeding

Zebrafish were maintained and bred at standard temperature conditions of 28 °C. All zebrafish experiments were performed according to the guidelines issued by the Institutional Animal Ethics Committee (IAEC) of the CSIR-Institute of Genomics and Integrative Biology, India. AB (WT), *Tg*(*actin:eroGFP*), *Tg*(*actin:cyroGFP*), and *Tg*(*actin:ERroGFPiE*) were used in this study.

### 2.2. Cloning of roGFP Constructs

Conventional as well as homologous recombination-based cloning was done for generating the constructs to be used for transgenic lines. pSS550 was used as the parent plasmid for the cloning and was a kind gift from Dr. Sridhar Sivasubbu. roGFP2 and roGFPiE were amplified from pRSETB-ro2 and pQE30-ro1/iE obtained as a kind gift from Dr. James Remington’s lab. *Tg*(*actin:eroGFP*) was made from the construct made by restriction digestion-based method, where 3 primers (no. 1 to 3) were used to add Hspa5 signal sequence upstream of roGFP2. Primers 4 and 5 were used to amplify the final construct and were subcloned into the pSS550 vector using restriction digestion sites for NheI and ClaI. For generating *Tg*(*actin:cyroGFP*), roGFP2 was amplified from pRSETB-ro2, with primers 6 and 7 having 15 bp sequence homology to the vector ends obtained after restriction digestion. For *Tg*(*actin:ERroGFPiE*), roGFPiE was amplified and 78 bp corresponding to the Hspa5 sequence signal of 26 aa were added by sequential PCR using primers 8, 9 and 10 and 11 and 12. All primer sequences are provided in Supplementary Table S1. In case of ER transgenic lines, the reverse primer had a KDEL sequence as well, which is an ER retrieval signal. Each of these, the vector, and the insert, were then recombined using an In-Fusion HD^®^ cloning kit (Takara Bio USA, Inc., Mountain View, CA, USA), where a 15 min reaction at 50 °C was setup with enzyme mix provided in the kit. For all the 3 clonings, the reaction mix was transformed in lab-made DH5α-competent cells and colonies were screened the next day.

### 2.3. In Vitro Transcription

Tol2 transposase was in vitro transcribed using pCS2, where it was first linearized by using restriction enzyme NotI. The transcript was made using SP6 RNA polymerase using the mMessage mMachine kit from Invitrogen with 1 µg of template DNA. The plasmid itself had polyA at the 3′ end, so addition of polyA tailing step was not required. The quality of RNA was assessed using agarose gel electrophoresis and stored at 80 °C until further use.

### 2.4. Injection and Generation of Transgenic Lines

One-cell stage embryos were collected after 15–20 min of removing the dividers. The respective roGFP construct and tol2 transposase mRNA were reconstituted in such a way that 1 nL contained 12.5 pg of each, and hence 1 nL of the volume was injected in one-cell stage embryos of WT background. Water was changed in the evening, and GFP-positive embryos were screened at 2 or 3 dpf stages using a ZEISS AxioScope A1 microscope (with Axiocam HRc^®^). At least 100 GFP-positive embryos were put into adults to grow for each line. After 3 months, progeny from three independent founder lines (obtained by backcrossing with the WT line) were grown. Embryos obtained from at least the F2 stage, or more were used for experiments. Before each experiment, 1 dpf embryo was screened for GFP fluorescence using ZEISS AxioScope A1 microscope and positive embryos were processed.

### 2.5. Zebrafish Single Cell Suspension and Primary Culture

GFP-positive (obtained from the transgenic lines) and WT embryos were first dechorionated manually and transferred to 1.5 µL microcentrifuge tubes (MCTs). Fifty and 100 embryos were taken for imaging and Western blotting experiments, respectively. After dechorionation, deyolking was done by adding 100 µL of lab made ringer’s solution, constituted as described in ‘The Zebrafish book’ (116 mM NaCl, 2.9 mM KCl, 5 mM HEPES, pH 7.2), for 5 min with intermittent pipetting with 200 µL tip [38]. Then, 1 mL of 1X trypsin-EDTA solution (GIBCO), pre-warmed at 29 °C, was added and kept at 29 °C in a dry bath. Additionally, 27 µL of 100 mg/mL collagenase type IV (GIBCO) was also added to it. Pipetting up and down was done using 1 mL tip for disruption of embryos into cells after every 5 min for a total incubation time of 20 min. The reaction was stopped using 200 µL FBS, kept for 2 min and the suspension was spun at 400× *g* for 5 min. Cells were suspended in L15 media (Sigma- Aldrich, St. Louis, MO, USA) with 10% FBS after 2 washes with chilled 1X PBS. Cell suspension was seeded either in 8-chambered coverglasses or 6-well cell culture plates (precoated with 0.2% gelatin) for imaging or Western blotting, respectively. The primary culture contains all cells from embryos which could attach to the substratum. The media was changed the next day, and the culture was processed for further experiments.

### 2.6. Localization Using Organelle Trackers

First, the media was removed and 10 µg/mL of Hoechst solution, made in 1X HBSS (Hank’s Balanced Saline Solution; GIBCO), was added to the primary culture in chambered slides. After 15 min, organelle trackers, working solutions made in 1X HBSS were added for 30 min. Additionally, 10 µM ER-tracker, 1 µM of each Mito and LysoTracker (Invitrogen, Carlsbad, CA, USA) were used. Two washes were given with 1X HBSS and the L15 media was added again. Imaging was done using 63X oil objective, in 4 zoom on a Leica SP8 confocal microscope.

### 2.7. Protein Extraction and Western Blotting

Embryos were harvested by removing water from the Petri plates and transferred to 1.5 mL MCTs. Approximately 39 embryos were taken for Western blotting experiments. Then, 120 µL of NP40 buffer (Invitrogen; 50 mM Tris, pH 7.4, 250 mM NaCl, 5 mM EDTA, 50 mM NaF, 1 mM Na_3_VO_4_, 1% Nonidet P40 (NP40) 0.02% NaN_3_), with PICs (Protease and Phosphatase inhibitor cocktails; Sigma), was added to the embryos followed by homogenization using tissue homogenizer (Motor-Driven Tissue Grinder; BR Biochem, New Delhi, Delhi, India). Spin was given at 16,000× *g* for 15 min and supernatant was collected. For protein extraction from primary culture cells, cells were scraped off in PBS and collected in 1.5 mL MCTs. NP40 buffer with PICs was added and the cell suspension was vortexed for 30 min at 4 °C. Spin was given at 16,000× *g* for 15 min and supernatant was taken. Protein concentration was estimated using a BCA kit (Pierce™ BCA Protein Assay Kits; Thermo Scientific, Waltham, MA, USA). Samples were prepared for the SDS page in laemelli buffer with 30 μg of protein per sample per gel. The 10–15% gels were made using premixed Acrylamide:Bisacrylamide (Liqui-gel, 29:1; MP Biomedicals, Santa Ana, CA, USA) solution according to the need. Wet transfer of proteins was done on 0.2 μ nitrocellulose membrane (Biorad, Hercules, CA, USA) at 70 V for 3 h. Membrane blocking was done with 5% BSA (Bovine Serum Albumin) in TBST (Tris-Buffered Saline with 0.2% Tween-20) and antibody dilutions were made in 2% BSA in TBS. Probing was done with α-GFP [39] (1:10,000; rabbit, Abcam, Cambridge, Cambs, UK), α-GRP78 [40] (1:2000; rabbit, Protein tech, Rosemont, IL, USA), α-GRP94 [41] (1:1000; goat, Enzo Life Sciences, Farmingdale, NY, USA), α-Actin [42] (1:2000; mouse, Sigma- Aldrich, St. Louis, MO, USA), α-CoxIV [43] (1:1000, rabbit, Cell Signaling Technology, Danvers, MA, USA), and α-SOD2 [44] (1:1000, rabbit, Abcam, Cambridge, Cambs, UK) antibodies. Blots were developed using Crescendo solutions (Immobilon^®^ Crescendo Western HRP Substrate; Millipore, Burlington, MA, USA) in Syngene gel imager (Syngene G: box F3, Bengaluru, Karnataka, India).

### 2.8. Bacterial Expression and Purification of roGFP2 and roGFPiE

Bacterial expression plasmids pRSETB-roGFP2 (6xHIS, *Amp^+^*) and pQE30-roGFP1-iE (6xHIS, *Amp^+^*), received as a kind gift from Sir James Remington, were used for purification of roGFP2 and roGFPiE, respectively. The plasmids were transformed into *E. coli* BL21 (DE3) and single colonies were inoculated. The primary inocula were used to inoculate 1 L LB with Ampicillin (100 µg/mL, MP Biomedicals, Santa Ana, CA, USA) bulk cultures. Protein expression was induced with 0.7 mM IPTG (Isopropylthio-β-galactopyranoside; MP Biomedicals, Santa Ana, CA, USA) upon reaching OD of 0.6. After induction, cultures were incubated for 18 h at 30 °C, 200 rpm shaking speed. The cells were harvested and resuspended in a pre-chilled base buffer (1X PBS + 5% glycerol) containing 2 mM PMSF (Phenylmethylsulfonyl fluoride; MP Biomedicals, Santa Ana, CA, USA). Cell lysis was done using sonication and the lysates were centrifuged at 17,000× *g* for 1 h at 4 °C. The supernatants thus obtained were loaded onto separate gravity-assisted Ni-NTA agarose affinity chromatography columns (Merck Millipore, Burlington, MA, USA) pre-equilibrated with chilled base buffer. The columns were washed with 10 column volumes of base buffer containing 5 mM imidazole (MP Biomedicals, Santa Ana, CA, USA). Proteins were eluted with 2 column volumes of base buffer containing 500 mM imidazole. The imidazole was removed by buffer exchange with the base buffer at 4 °C in Amicon Ultra-15 filter unit NMWL 3 kDa (Millipore, Burlington, MA, USA).

### 2.9. Redox Titration of roGFP2 and roGFP1-iE

Based on previous protocols, roGFP2 and roGFPiE were titrated in GFP redox buffer (75 mM HEPES + 125 mM KCl + 1 mM EDTA, pH 7.4) containing 0–10 mM DTT (DL-dithiothreitol, MP Biomedicals, Santa Ana, CA, USA, as a reducing agent) and 10–0 mM diamide (Sigma- Aldrich, St. Louis, MO, USA; as an oxidizing agent) in a reciprocal manner with steps of 1 mM initially for standardizations. Then, 10 concentrations of reducing:oxidizing agents were taken between 4 and 6 mM. The titration was set up in a black opaque 96-well plate (compatible with fluorescence measurement). The final volume of the redox reaction was kept at 100 µL and the final concentration of GFP at 10 µM. The mixture was incubated in the dark for 1 h at room temperature. Finally, the fluorescence intensity of respective wells was measured in TECAN Infinite M200 Pro ELISA plate reader (Morrisville, NC, USA, operated by TECAN I Control V3.3.10.0 software). The excitation wavelength was set at 405 nm and 480 nm with the emission wavelength kept constant at 520 nm. The aim was to determine the relative proportions of DTT (reducing agent) and diamide (oxidizing agent) required to induce the transition of roGFP between its reduced and oxidized states, as monitored by a change in excitation wavelength. We found that somewhere between 5 and 6 mM of DTT, the flip happens. The 4–6 mM range was expanded to pinpoint the exact conc., and we found that at 5.4 mM, the flip is complete. The data was plotted with a 405/488 ratio on the *Y*-axis and conc. of DTT on the *X*-axis in Microsoft Excel.

### 2.10. DCFDA Assay for ROS Estimation

ROS estimation was done in a 96-well plate using the cell-permeant 2′,7′-dichlorodihydrofluorescein diacetate (H_2_DCFDA) (Thermo Scientific, Waltham, MA, USA) dye, and each well had 3 embryos. Twelve of the wells were used for one replicate of each line, so one replicate represents data from 36 embryos. Embryos in wells with 10 mM H_2_O_2_ were used as a positive control. Then, 200 μL of water was added to each well and a before reading was taken. Then, excess water was removed and 200 μL of 2.5 mM DCFDA was added to each well, incubated at 37 °C for 2 h, and readings were taken in TECAN Infinite M200 Pro ELISA plate reader at 488 nm. Data was analyzed using Microsoft Excel. An unpaired Student’s *t* test was done with Welch correction using Graph pad Prism 9.4.1.

### 2.11. Drug Treatments

Tunicamycin (Sigma) was used at 6 and 10 μg/mL for 8 h in primary cells according to the need for imaging as well as Western blotting experiments. A total of 0.5 mM DTT (Sigma) treatment for 8 h was given to embryos for Western blots while 10 mM DTT was given, and cells were immediately imaged for redox readings within 1 min.

### 2.12. Redox Imaging

Embryos were molded first in low-melting agarose and then redox imaging was done using a Leica SP8 confocal microscope with 10× objective, while 63× objective, zoom 4, was used for cells. First, the low-melting agarose (Invitrogen, Carlsbad, CA, USA) was heated to boil and kept at 36 °C in a dry bath for equilibration. Embryos were anesthetized using 0.004% Tricaine (Sigma-Aldrich, St. Louis, MO, USA) for 1 min or until they stopped moving, put in agarose for equilibration for a few seconds and immediately put as a drop on a rectangular coverslip. Embryos were checked for normal appearance and viability; however, heartbeat was not recorded due to technical limitations. Embryos were molded in the lateral position or according to the need using ZEISS light microscope (Trinuculor Stereo zoom microscope with digital camera- DX10 cam; Zeiss stemi 305, White Plains, NY, USA).

Sequential scan setting for imaging was used in a confocal microscope for redox imaging. In brief, there were four settings at which embryos were imaged, called channels. For embryos, channel 0: excitation (ex): 488 nm, emission (em): 505–540 nm; channel 1: bright field; channel 2: ex: 405 nm, em: 440–480 nm; and channel 3: ex: 405 nm, em: 505–540 nm. For cells, the same settings were used except emission collected for excitation at 488 nm and 405 nm in channel 0, and 3 was from 505 to 530 nm. The obtained data was processed for image analysis. Channels were changed between frames for imaging. Here, channel 2 readings were procured for subtraction of background fluorescence.

### 2.13. Image Analysis

Images were extracted from .lif (format of Leica microscope) files in .tiff format as grayscale images. These were processed in Python 3 using in-house generated script. The pseudo code of Python 3 script is as written in the steps below:

1. Three frames of images are read (image1 = 505/488, image2 = 505/405, image3 = 450/405 (emission nm/excitation nm)).
2. The intensity of a pixel is calculated as an average of the surrounding pixels (1 pixel width) in the x y plane for image1 and image2.
3. All pixels where the fluorescence in image1 < CUTOFF are replaced by zero in image1 and image2.
4. All pixels where the fluorescence in image3 > BACKGROUND_CUTOFF are replaced by zero in image1 and image2.
5. The ratio of image1/image2 is saved as the ratio file containing the ratio information for each of the pixels.
6. The pixels are colored according to the criteria set in the different conditions. The Ratio-2 images obtained were viewed in the 3D viewer in ImageJ v1.54r.

## 3. Results

### 3.1. Generation and Characterization of Cytosol and ER-Targeted roGFP Transgenic Lines

**Figure 1:**
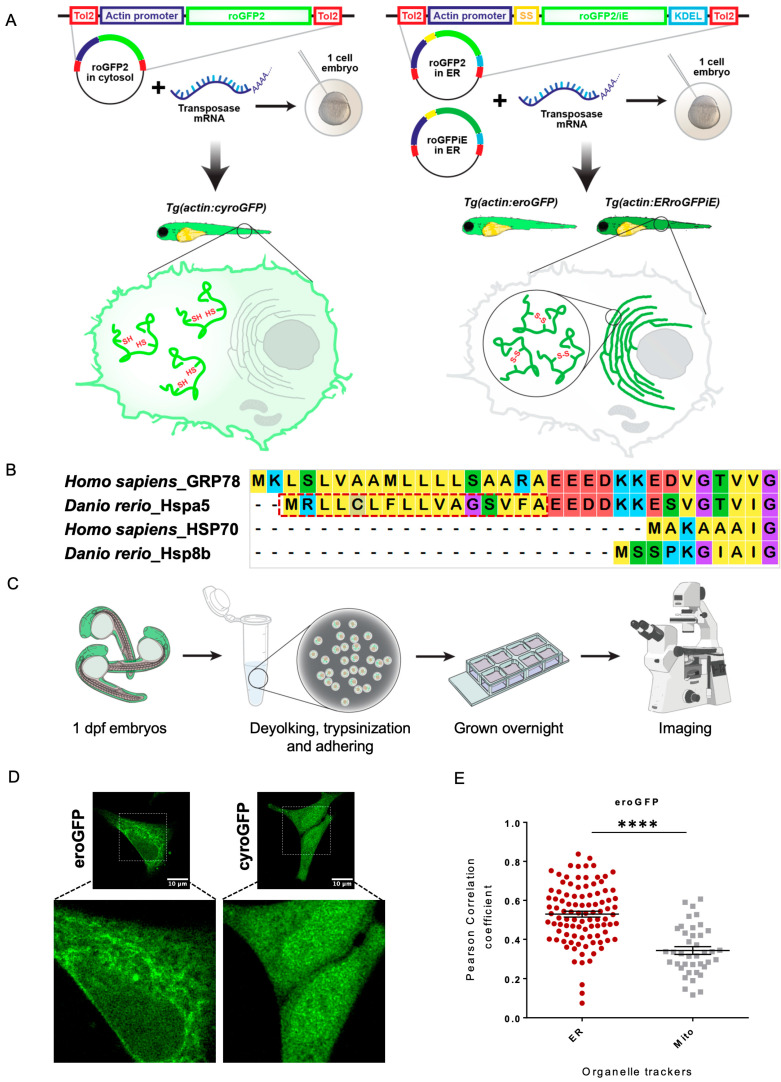
roGFP2 is localized in the endoplasmic reticulum in the eroGFP transgenic line. (**A**) A schematic representation of the construct and strategy used to make the transgenic lines, where roGFP2 was targeted to either cytosol (depicted as green cytosol) or ER, and roGFP1/iE to ER (depicted as green ER network). The construct consisted of actin promoter driving the roGFP2/roGFP1/iE flanked by tol2 sites on either side for correct transposase-mediated recombination. Constructs along with transposase mRNA was injected in the one cell stage embryos to generate the transgenic lines. The lines are named according to the zebrafish convention of nomenclature. (**B**) Amino acid (aa) sequence alignment of Hspa5 (GRP78) and Hsp8b (HSP70) from zebrafish and human. Alignment was done using MEGA (https://www.megasoftware.net/, accessed on 1 June 2026) to find the ER targeting signal sequence specific to Hspa5 and not present in the cytosolic Hsp8b. Dotted red box shows the 16 aa used in this study for targeting roGFP2 to the ER. For ERroGFPiE line, 26 aa were taken. (**C**) A schematic depicting the procedure of generating primary cell culture from zebrafish embryos. Briefly, the single cell suspension from entire embryos was grown in L15 media at 28 °C in chambered cover glasses followed by imaging next day. (**D**) Fluorescence images of primary cells made from 1 dpf zebrafish embryos (*n* = 100 embryos per line), showing roGFP signal in eroGFP (left panel) and cyroGFP (right panel) transgenic lines. Images in the bottom panel show enlarged areas of the cells in the top panel (confocal microscope, 63× objective, 4× zoom, scale 10 µm). (**E**) Scatter plot shows Pearson correlation coefficient for colocalization of green and red channel for eroGFP line, where each dot corresponds to one cell (*n* = 3 biological replicates from 100 embryos in each replicate). Significance was tested using two-tailed Student’s *t* test with unequal variance, where **** is *p* value < 0.0001. Abbreviations: dpf—days post fertilization, ER—endoplasmic reticulum, mito—mitochondria.

To investigate if the redox potential of the cytosol and the ER varies across different tissues of zebrafish, we generated zebrafish transgenic lines expressing roGFP2 either in the cytosol or the ER (Figure 1A). The roGFP2 sensors have disulfide bonds engineered in the proteins such that the protein maintains different conformations in reducing and oxidizing milieus. Both the conformations absorb energy at 405 and 488 nm of light and emit at 505 nm; however, the ratio of absorbance at these two wavelengths shifts according to the redox environment in which it is present. Absorbance at 405 is higher when the probe is oxidized (therefore, the emission at 505 is higher when excited at 405 nm), while absorbance at 488 nm increases when it is in a reduced state [4]. So, the values obtained for emission at 505 nm when excited at 488 nm divided by the emission at 505 nm when excited at 405 nm (488 nm/405 nm) acts as a surrogate for the redox status. Fusing roGFP2 to proteins like Grx1 is known to increase the time resolution of recording changes in redox potential [3,45]. These fused probes equilibrate faster with GSH:GSSH-dependent redox potential and are beneficial (and sometimes necessary) for time kinetics experiments. However, we did not want to measure kinetics in this study, so we used native roGFP probes.

roGFPiE is a redox sensor engineered to sense relatively oxidizing compartments like the ER [46], so we made another transgenic line targeting roGFPiE to the ER. We generated three different zebrafish transgenic lines expressing roGFP2 in the cytosol, *Tg*(*actin:cyroGFP*) (hereafter cyroGFP) and ER, *Tg*(*actin:eroGFP*) (hereafter eroGFP), and roGFPiE in the ER, *Tg*(*actin:ERroGFPiE*) (hereafter ERroGFPiE). The actin promoter ensured ubiquitous expression of GFP in the developing embryos (Figure S1A). The default localization of the roGFP2 expressed would be in the cytosol. However, to target the sensor to the ER, we needed to fuse an ER-targeting signal sequence to the N-terminus of roGFP2. No previous study has targeted exogenous proteins to the ER in the zebrafish and thus the ER-targeting signal for zebrafish was not known. We chose to identify the sequence from GRP78 or Bip, a canonical ER chaperone whose targeting signal sequence is routinely used to target proteins to the ER in *S. cerevisiae* and mammalian cells [47,48]. We aligned the protein sequences of GRP78 and cytosolic chaperone HSP70 from *Homo sapiens* to that of the *D. rerio* Hspa5 and Hsp8b (HSP70) to identify the putative ER signal sequence. We selected the first 16 amino acids (aa) that appeared to be specific to the ER-targeted Hspa5 and were not present in its cytosolic counterpart, Hsp8b (Figure 1B). These 16 aa of Hspa5 were fused to the N-terminal of roGFP2 and the first 26 aa of Hspa5 were fused to the N-terminal of ERroGFPiE to assess the minimal sequence needed for targeting (Figure 1B). In order to look for the efficiency and cleavage site prediction, we used the SignalP 6.0 tool (https://services.healthtech.dtu.dk/services/SignalP-6.0/) (accessed on 1 June 2026) for both of the signal peptides [32]. We found that signal peptide cleavage site prediction was after 16th aa in both cases (Figure S2A,B). Thus, the design principles of the signal sequence were, in principle, sufficient to target the protein to the ER.

**Figure 2:**
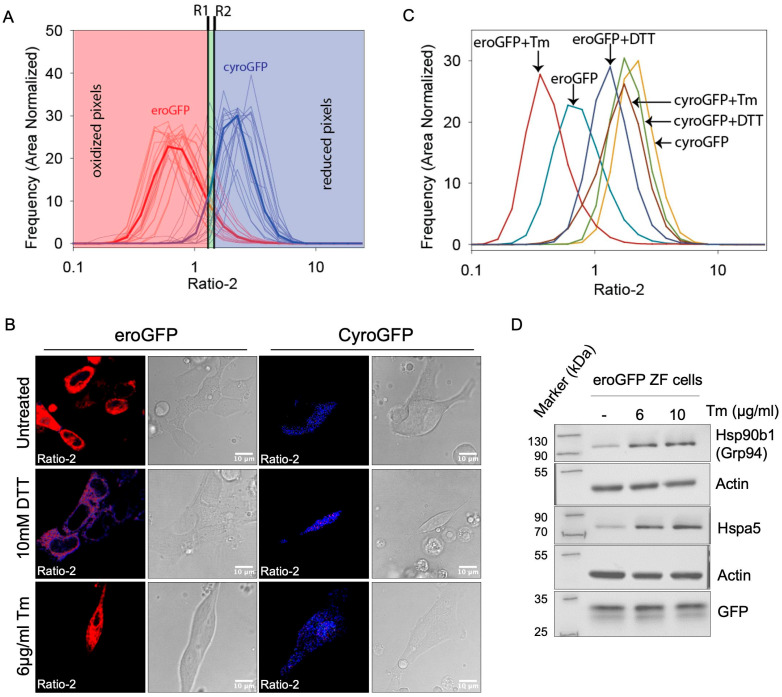
Primary cells from roGFP transgenic lines are sensitive to chemical perturbations. Primary cells from 1 dpf embryos were grown overnight and 6 and 10 μg/mL of tunicamycin (Tm) treatment was given for 8 h. (**A**) Both untreated and treated cells were imaged to determine redox potential using confocal microscope. Raw images were then analyzed by a Python 3 script to calculate pixel-wise Ratio-2 values. Frequency of Ratio-2 values was area-normalized for untreated cells and graph was plotted. The pixels were divided into 3 bins according to the values obtained and given colors on RGB scale (Red–Green–Blue). R1 is the boundary defined as 1 standard deviation away from mean eroGFP value while R2 is 1 standard deviation less than mean cyroGFP value. Ratio-2 values below R1 were marked red (oxidizing), between R1 and R2 marked green (intermediate) and higher than R2 were marked blue color (reducing). (**B**) Cells were treated with either 6 µg/mL of Tm (8 h of treatment) or 10 mM DTT (immediately imaged after treatment). Images represent maximum projection images of untreated 10 mM DTT and 6 µg/mL Tm-treated cells from left to right, respectively (Confocal microscope, 63× objective, zoom 4, scale 10 µm). (**C**) Ratio-2 values are plotted for each of these conditions. Each line represents mean Ratio-2 for each treatment (SET1), done on one day and it was repeated thrice. N is variable in each condition and contains value from at least 6 fields. (**D**) Western blots show expression of GRP78 (Hspa5) and GRP94 (Hsp90b1) as ER stress markers, actin was used as a loading control and GFP was probed to show expression of the transgenic protein. Abbreviations: BF—bright field, DTT—dithiothreitol.

In the embryos, where cells are packed together, it was difficult to visualize the organelles clearly. Thus, we prepared a primary cell culture from 1 dpf (days post fertilization) zebrafish embryos by dissociating the cells from the embryo and allowing them to attach to a substratum (Figure 1C). The GFP fluorescence in the cyroGFP cells was present throughout the cell, reminiscent of its localization in the cytosol (Figure 1D and Figure S3A). The GFP fluorescence in the eroGFP showed a tubular pattern distinct from the cyroGFP and we observed a perinuclear distribution of the GFP with nuclear exclusion (Figure 1D and Figure S3A). We co-stained the cyroGFP, eroGFP and ERroGFPiE cells with various organelle trackers to visualize organelles clearly (Figure S3A). Visual analysis showed a similar pattern of roGFP and ER-Tracker Red in the eroGFP line, whereas the MitoTracker Red and Lyso-Tracker Red had very different staining patterns to roGFP (Figures S3A and S4A–H). This was confirmed by quantification of the Pearson correlation coefficient for colocalization of roGFP with ER and MitoTracker Red in eroGFP cells, which revealed significant correlation with ER Tracker Red (Figure 1E). Similar trends were observed for the ERroGFPiE line, indicating ER localization (Figure S3A,B). Thus, it appears that both 16 aa and 26 aa can target proteins to the ER and that the 16 aa ER-targeting signal sequence is sufficient. Further, eroGFP cells also showed an oxidizing environment when checked for redox status, again confirming the ER localization of roGFP (Figure 2A–C). Quantification of roGFP in the cyroGFP line did not show any colocalization with ER or MitoTracker Red (Figure S3C). Taken together, the transgenic lines show redox-sensitive GFPs localized correctly in their respective compartments.

### 3.2. The eroGFP Line Does Not Exhibit ER Stress

Overexpression of exogenous proteins can perturb protein homeostasis and stress the cell. Hence, we assessed the eroGFP lines for ER stress marker expression. The expression of GRP78 (Hspa5 in zebrafish), known to be upregulated during ER stress [49], did not change significantly in the eroGFP line compared to WT (Figure S5A,B). Manganese superoxide dismutase (SOD2) protects cells against oxidative stress [50,51]. Our studies showed no significant change in Sod2 expression in the transgenic lines (Figure S5A,C). An increase in disulfide bond formation in the ER due to overexpression of proteins may also result in an increase in the reactive oxygen species (ROS) levels [52,53], especially since eroGFP harbors engineered disulfide bonds [4]. We used the DCFDA assay to detect ROS levels and found it to be comparable between the eroGFP and the control embryos (Figure S5D). Thus, we concluded that the eroGFP transgenic lines did not show any signs of ER stress in normal conditions.

### 3.3. The roGFP Lines Respond to External Perturbations in Cellular Homeostasis

For the roGFP transgenic lines to be useful for monitoring redox potential in the animal, the roGFP proteins must be responsive to changes in the oxidative state. We measured the emission peak of the roGFP when excited at 405 nm and 488 nm with live emission scans of 4 dpf larvae on the confocal microscope. We found the expected 505 nm peak when excited at 405 nm or 488 nm in all transgenic lines (Figure S6A–E). The head region in eroGFP embryos showed a peak at 505 nm upon excitation at 405 nm or 488 nm, indicating that roGFP is oxidized in the ER, while cyroGFP embryos showed very minimal 405 signal (505 nm emission with 405 nm excitation), suggesting a reducing milieu (Figure S6A,C). Similar results were also obtained for the trunk region of the larvae (Figure S6B,D). Thus, roGFP2 targeted to ER and cytosol reported the expected relative redox differences between the two compartments. The transgenic line ERroGFPiE also showed two characteristic peaks where the emission signal at 405 nm was higher than 488 nm, as previously reported for this variant (Figure S6E) [46]. The oxidized form of roGFPiE is predominant in the ER (405 peak in Figure S6E). These peaks were absent in the WT zebrafish embryos (Figure S6F), showing the signals to be specific for the fluorescent reporters.

To assess the response of the roGFP lines to perturbations in cellular redox potential, we used dithiothreitol (DTT) and tunicamycin (Tm) treatment. To avoid variability due to drug penetration in the embryo, we prepared a primary culture of cells dissociated from 1 dpf embryos. The cells were treated either with 10 mM DTT (reducing reagent) or 6 µg/mL and 10 µg/mL of Tm (ER stress inducer). To test the efficacy of Tm in inducing ER stress in the primary culture cells, we collected protein from the cells after 8 h of treatment and performed Western blots for ER stress markers. We found upregulation of both Hspa5 and Hsp90b1 (GRP94) (canonical ER stress markers) in the eroGFP cells at both the concentrations of Tm (Figure 2D). All further perturbation experiments were performed after 8 h of treatment with 6 µg/mL of Tm. All DTT treatments were done with 10 mM DTT, and imaging was started within 1–2 min of addition of the compound.

Two different fluorescence measurements were performed for both the eroGFP and cyroGFP probes: fluorescence emission from 505 to 540 nm upon excitation at 488 nm and from 505 to 540 nm upon excitation at 405 nm. The fluorescence ratio of these two measurements at 488 nm/405 nm was then obtained after analysis and will be henceforth referred to as Ratio-2. Pixel-wise frequency distribution of Ratio-2 in eroGFP and cyroGFP cells showed that they followed a roughly log-normal distribution (Figure 2A). We transformed the ratio in log2 scale and set two boundaries for quantitative demarcation of reduced and oxidized regions. R1 boundary was defined as one standard deviation more than the mean eroGFP value, while R2 was set at one standard deviation less than the mean cyroGFP value. Pixels with Ratio-2 less than R1 represented ‘oxidized’ pixels and those with Ratio-2 greater than R2 represented ‘reduced’ pixels. Pixels between R1 and R2 represented the intermediate between the oxidized and reduced state (Figure 2A). For ease of visualization in the cellular and embryonic images, the oxidized pixels were false-colored red, the reduced pixels were colored blue and the intermediate pixels green.

We determined the Ratio-2 in the transgenic cells treated with DTT. Treatment of eroGFP cells with DTT resulted in an instant and significant increase in Ratio-2, indicating a change from an oxidized to reduced state (Figure 2B,C). Tm was expected to cause ER stress and induce reducing conditions in the ER [47]. Later, the work by the same group reported a reflux of roGFP to the cytosol upon Tm treatment as being the reason for the observed reducing signal [54]. Instead, treatment with Tm decreased the Ratio-2 in eroGFP, marginally suggesting a shift to an oxidized state in our study (Figure 2B,C and Figure S7). This trend towards a decrease in Ratio-2 when treated with Tm was not consistent across different sets of cells (Figure S7); importantly, the ER redox potential did not show a shift towards a reducing state upon treatment with Tm. Importantly, there was no redistribution of eroGFP from ER to the cytosol. This data also suggests that reflux of roGFP is not prominent in our model. The cyroGFP cells did not show any significant change in the Ratio-2 in response to DTT or Tm; likely because the cytosol is already reduced (Figure 2B,C and Figure S7). Taken together, the eroGFP and cyroGFP zebrafish lines were able to report the expected redox potentials and respond to external perturbations.

### 3.4. Redox Potential of ER and Cytosol Show Region-Wise Differences

**Figure 3:**
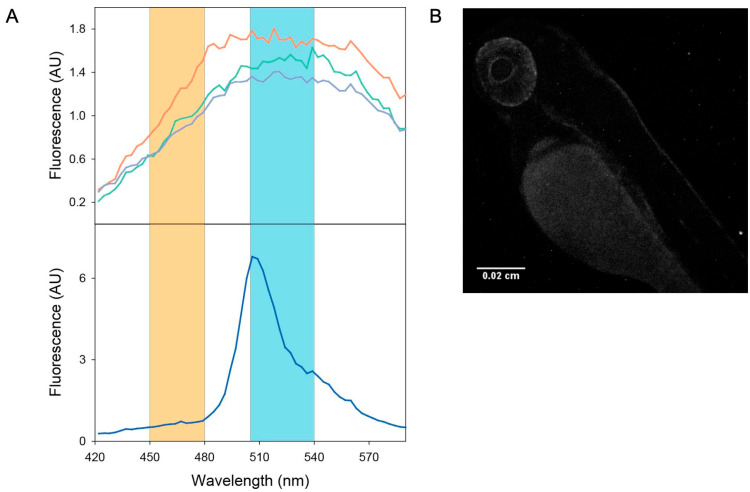
Determining autofluorescence signals from a WT embryo. (**A**) The 3 dpf WT embryo lacking any roGFP expression was anesthetized, mounted in lateral position, and excited at 405 nm and emission scan was taken from 422 to 593 nm to determine autofluorescence signals (upper panel). Readings were taken at every 3 nm interval, and the graph was plotted. Three lines represent three individual embryos. Similarly, emission scan was taken for 1 µM roGFP2 solution at same settings (lower panel). Shaded light yellow color region depicts emission range of autofluorescence while light cyan blue indicates roGFP signal (**B**) Embryos were anesthetized, mounted in agar in lateral position and imaging was done with excitation at 405 nm and emission was collected from 440 to 480 nm. Representative autofluorescence signal from one WT embryo is a maximum projection image adjusted with ImageJ (Confocal microscope, 10× objective, scale—0.02 cm). Abbreviations: dpf—days post fertilization.

Obtaining a quantitative redox map of zebrafish embryos was fraught with hurdles. High autofluorescence (scatter) in certain regions of the embryo precluded determination of the correct ratio of GFP fluorescence upon excitation at the two different wavelengths. To solve this problem, we needed to determine the contribution of autofluorescence in the different regions of the embryo. We took fluorescence images and collected emission from 505 to 540 nm upon excitation at either 405 nm or 488 nm, using WT zebrafish embryos with no roGFP2 expression. While excitation at 488 nm had negligible autofluorescence, 405 nm showed high autofluorescence in certain regions of the embryo. Since each embryo has unique variations in anatomical structure, it is impossible to create a universal quantitative map of autofluorescence in these embryos. To obtain the contribution of autofluorescence in the 405 nm excitation channel in the roGFP transgenic embryos, we needed to generate an autofluorescence map for each embryo. Deconvoluting autofluorescence for a fish with a roGFP2 probe would require us to determine the contribution of autofluorescence with a proxy measurement at a wavelength where the authentic roGFP2 fluorescence has negligible contribution. We found that WT embryos, when illuminated at 405 nm, showed an autofluorescence emission peak around 515 nm and exhibited a prominent shoulder between 440 nm and 480 nm (Figure 3A, upper panel). This shoulder was absent in the fluorescence peak of purified roGFP2 (Figure 3A, lower panel). Thus, autofluorescent regions in a roGFP embryo show significant emission (in the shoulder) between 440 nm and 480 nm while being excited at 405 nm. This was used to differentiate from true roGFP fluorescence. We therefore recorded an additional image of each embryo with 405 nm excitation and measured the emission from 440 to 480 nm (Figure 3B). This image allowed us to build a quantitative estimate of the autofluorescent regions of the embryo; these regions were excluded from all further analysis. We found that when we remove all pixels with more than 10 intensity values in the background channel, the ratio distribution and the median are independent of the autofluorescence value (Figure S8A,B). Additionally, comparison of autofluorescence and ratio images showed clearly that the regions of autofluorescence are effectively removed from the calculations (Figure S9). Thus, the pipeline effectively nullifies the contribution of autofluorescence and is negligible overall.

**Figure 4:**
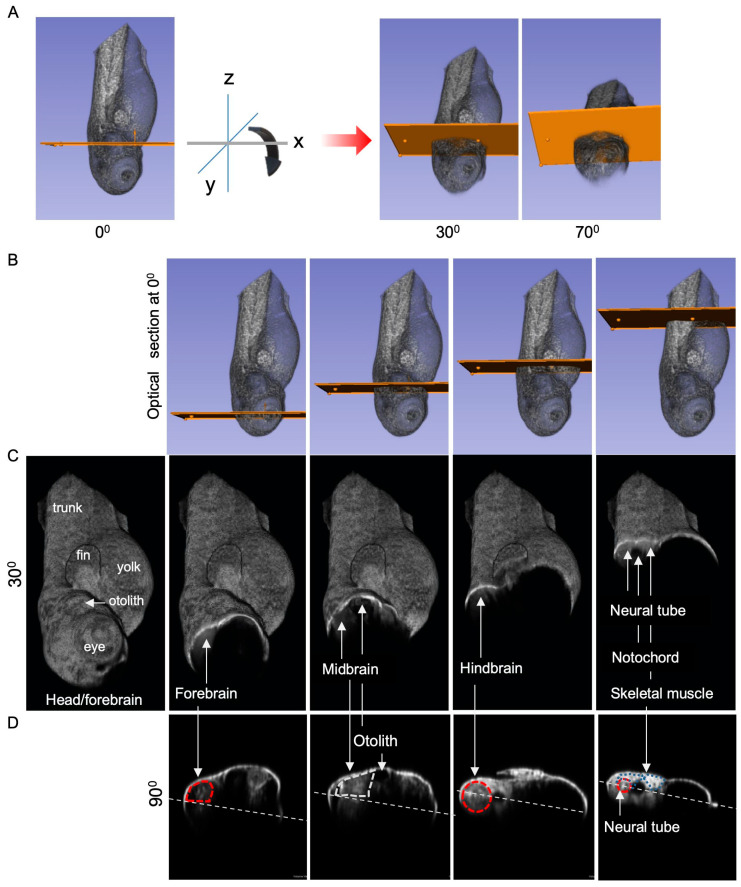
Representative cross sections and regions of interests marked on GFP fluorescence images of an embryo. (**A**) The left image shows the way the embryo was imaged, with yellow plane showing the region where optical sectioning was done to visualize the redox ratios inside the embryo. The middle and right images indicate the way the embryo was oriented (rotated) to 30° and 70°, respectively, also indicated by the arrow, making the yellow disk visible in the images. (**B**–**D**) Images show GFP signal of a 3 dpf embryo representing the rotation angles to show redox ratios in the subsequent Figures. Cross section at these positions remains similar for further Figures. (**B**) All images in the panel show the region from where optical sectioning was done. (**C**) All images in the panel show the GFP signal in the oriented image of embryo sectioned at that region. (**C**) Images in the panel from left to right show labeling of major regions, forebrain, midbrain, hindbrain, muscle, neural tube, and notochord (marked using white arrows), which become visible after optical sectioning is done at the places indicated and the embryo is rotated at 30°. (**D**) Images in the panel shows the same regions (as in **C**) when the embryo is rotated at 90°. White line in each image indicates the lateral line which will divide the embryo into left and right halves. Red dotted shapes mark forebrain, hindbrain and neural tube while white mark midbrain regions. (Confocal microscope, 10× objective). Abbreviations: dpf—days post fertilization.

Another problem we faced while imaging zebrafish larvae was visual access to the whole embryo under the confocal microscope. Due to the thickness of the tissue, we could capture only one of the lateral halves of the larva. As the tail region of the animal is thinner, we could image nearly the whole depth, but the head and trunk containing the visceral organs could be only partially imaged. All confocal scans were performed with the larvae oriented laterally. The 3D stacked images could then be rotated and viewed from various angles according to the requirement (Figure S10). Optical sections were then taken to look closely beneath the surface of the regions we found important. The particular section used in each image is indicated by the schematic of a plane slicing through the 3D-reconstructed image (Figure 4A).

Once the images were acquired and analyzed, it was a challenge to identify the precise anatomical structures showing interesting redox potential patterns due to a lack of reference points in the dark images. Thus, for the current study, we have focused our analysis on four virtual slices (1–4) that cover the broad regions of brain and muscle in the larvae (Figure 4B–D). We have observed many interesting regions of redox anomaly outside the brain and muscle which are not discussed here (the raw dataset is available at https://doi.org/10.7910/DVN/PRCFJM).

To determine the highest and lowest boundaries of Ratio-2, we measured the Ratio-2 for a solution of purified roGFP2 in either DTT or N,N,N′,N′-tetramethylazodicarboxamide (diamide) to obtain Ratio-2 for the fully reduced form or the disulfide-bonded form, respectively. While comparing the Ratio-2 distribution of the recombinant protein roGFP2 with the Ratio-2 of the eroGFP or cyroGFP embryos, we observed an inconsistency. The eroGFP embryos that should ideally be fully oxidized showed a ratio lower than the fully oxidized recombinant protein (Figure S11A). Similarly, cyroGFP, ideally fully reduced, showed a ratio lower than fully reduced roGFP2 protein (Figure S11A). This suggested that the Ratio-2 distribution might be skewed to a lower value due to the residual background intensity in the 405 nm excitation channel. The fluorescence of roGFP2 in the 405 nm channel for both eroGFP and cyroGFP lines is much lower than in the 488 nm channel. This makes the denominator (during Ratio-2 calculation) more sensitive to background noise (Figure S11C,D). Importantly, the fold difference between the fully reduced and fully oxidized forms of purified roGFP2 was similar to the fold difference in Ratio-2 between cyroGFP and eroGFP in the embryos (Figure S11B). This suggested that the peaks of eroGFP and cyroGFP from the embryo must correspond to the peaks for oxidized and reduced roGFP, respectively, in the context of embryos with non-zero background fluorescence. Henceforth, instead of using the theoretical maxima and minima for Ratio-2, we decided to use the empirically determined peaks as maxima and minima for all calculations. Additionally, bright field images of all the embryos used for representation of redox ratios for all the lines are displayed together in one Figure (Figure S12).

**Figure 5:**
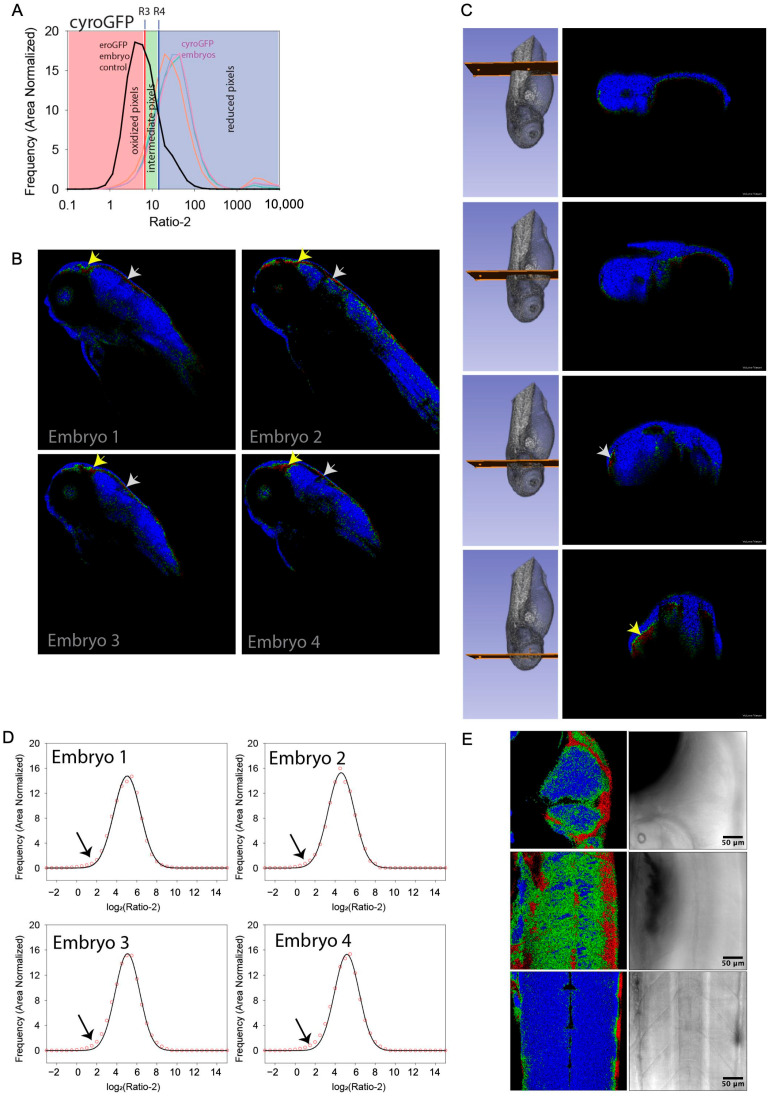
Redox status of cytosol is heterogeneous in zebrafish embryos. Three dpf live cyroGFP embryos were anesthetized, mounted in lateral position, and imaged by sequential scanning method, i.e., emission signal was collected from 505 to 540 nm first by excitation at 488 nm, followed by excitation at 405 nm. Autofluorescence signal was also collected, and images were analyzed to obtain redox ratios (more details in materials and methods). (**A**) The frequency of Ratio-2 values for cyroGFP and eroGFP lines were plotted to show the differences in the distribution of Ratio-2. R3 and R4 boundaries were set to define color scale where R3 is median of Ratio-2 from eroGFP embryo and R4 is one standard deviation from eroGFP median. Ratio-2 values below R3 were denoted as oxidizing and given red color. Ratios higher than R4 were denoted as reducing and given blue color while intermediate values between R3 and R4 were green. (**B**) Color-coded Ratio-2 values of four embryos depicted in RGB scale as embryo 1, 2, 3, and 4. (**C**) Left and right panel show GFP fluorescence with region of optical section and analyzed Ratio-2 images of a representative embryo, respectively. On the right panel, the first, second, third and fourth panel are images obtained after optical sectioning and embryo rotation to 90°. Yellow and white arrow indicates forebrain and hindbrain, respectively, in (**B**,**C**; scale bar 200 μm). (**D**) Frequency distribution of log_2_ values of Ratio-2 were fitted with a single peak Gaussian distribution. Red colored circle represents Ratio-2 values for all embryos and black solid line is obtained after Gaussian fit of the Ratio-2 values. The black arrow shows regions where oxidizing redox ratios are above the fitted black solid line. (**E**) Higher magnification (40×) images of a representative embryo show the head region (behind the eye; black region on top left), initial trunk (behind the yolk) and trunk regions (muscle; myotomes) from top to bottom while left and right are Ratio-2 and BF images, respectively (confocal microscope, 10× and 40× objectives; scale 40× BF image—50 µm). Abbreviations: dpf—days post fertilization, BF—bright field.

To be conservative in defining unexpected deviations in redox potential, we used the eroGFP as controls for cyroGFP. Since the ER-targeted roGFP is considered to be in a completely oxidized state, we picked the median of the eroGFP Ratio-2 as the lowest boundary (R3) for an oxidized state of cyroGFP. Thus, we defined any pixels with a Ratio-2 less than R3 as oxidized. Pixels with Ratio-2 higher than R4 (median + one standard deviation of eroGFP) was defined as reduced cyroGFP (Figure 5A). It is to be noted here that although the frequency distribution is quantitative in nature, the pixels in the different peaks cannot be resolved, as the peaks overlap. The thresholds are therefore somewhat arbitrary and hard; these therefore are only indicative of the nature of the redox potential. The color-based classification in this case, as well as the rest of the manuscript, is semi-quantitative at best, allowing us to comprehend the distribution of the redox potentials visually.

Once we applied all the above-discussed corrections to the analysis, we uncovered an interesting paradox. We found that the cytosolic redox potential was strongly oxidizing (sometimes as much as the ER) in multiple regions of the embryo (Figure 5B,C). This is supported by the frequency histogram (Figure 5D). Could these observations be spurious, or an artifact of the arbitrary division of a normal distribution? We think not, because of three reasons. One, the number of pixels in the lower range of Ratio-2 for cyroGFP was more than expected from a normal distribution obtained after fitting the curve for the top 75% of the Ratio-2 distribution (Figure 5D). Two, instead of being randomly distributed irrespective of anatomical regions, the oxidizing regions (red pixels) formed defined clusters in similar regions of the embryos, surrounded by intermediately oxidizing regions (green pixels) (Figure 5B,C). We confirmed the clustering by confocal imaging at a higher magnification (Figure 5E). Three, the oxidizing and the intermediately oxidizing regions were consistent between different embryos (Figure 5B). Thus, cyroGFP could delineate regions of the embryo with oxidizing cytosolic redox potential. Closer examination of the images (at higher magnification; 40×) revealed that the highly oxidizing regions of the embryo were absent around the skeletal muscles and were enriched in areas surrounding the midbrain and forebrain regions (Figure 5B,C,E). This was true for multiple 3 dpf embryos (Figure 5). Close observation also revealed that the oxidizing regions formed a layer surrounding the ‘normal’ reducing regions deeper in the brain (Figure 5E). From our data, it is not clear whether the oxidizing layer is within the brain tissue or is a layer outside of the brain.

### 3.5. Map of the Redox Potential of ER Using eroGFP and ERroGFPiE

**Figure 6:**
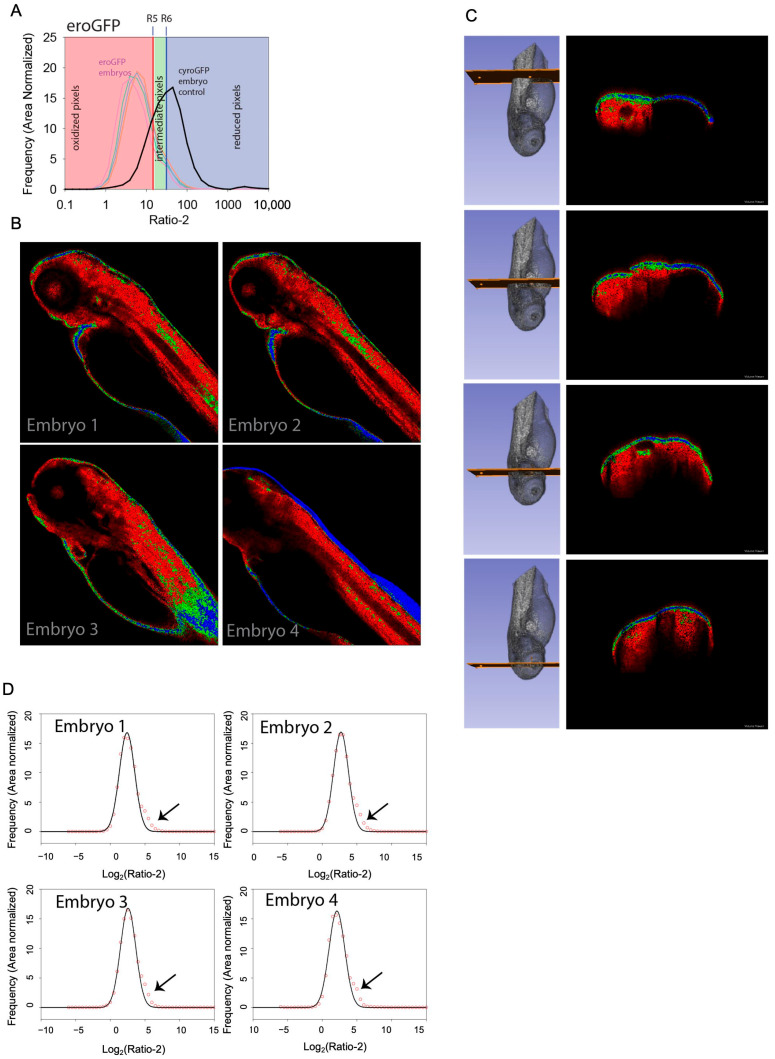
Redox state of ER differs across regions of zebrafish embryos. The 3 dpf live eroGFP embryos were anesthetized and imaged to obtain redox status. Pixel-wise ratios were calculated to obtain Ratio-2 values using Python 3. (**A**) log_2_ of Ratio-2 values of eroGFP embryos were plotted to obtain the frequency distribution and define color scale with cyroGFP embryo as control. R5 and R6 boundaries were defined where R6 is median of cyroGFP embryo and R5 is one standard deviation lower than cyroGFP median. For color coding, pixels below R5 and higher than R6 were given red (oxidizing) and blue (reducing) colors, respectively, while the intermediate pixels were colored green. (**B**) Color-coded Ratio-2 images of four embryos are shown separately. (**C**) Left and right panels show GFP fluorescence with region of optical sectioning and analyzed Ratio-2 images, respectively. First, second, third and fourth panel from top to bottom show the Ratio-2 images sectioned at the indicated positions and rotated to 90°. (**D**) log_2_ values of Ratio-2 were plotted against frequency with area normalization where red circles are the actual calculated values while black solid lines are fitted values. The graphs were made for each embryo individually. Black arrow indicates the real Ratio-2 values above the fitted data (Confocal microscope, 10× objective; scale bar 200 μm in **B**,**C**). Abbreviations: dpf—days post fertilization.

For analyzing eroGFP and for creating a conservative map of unexpected deviations in redox potential in the ER, we used the cyroGFP peaks as controls. We defined the median Ratio-2 of cyroGFP (R6) as the boundary for identifying anomalous reduced pixels in the eroGFP embryos. Pixels with Ratio-2 less than R5 (one standard deviation lower than the median of cyroGFP) were defined as regions with eroGFP in an oxidized state. It must be noted here that the boundaries (R5, R6) defined for eroGFP are distinct from the boundaries defined for cyroGFP (R3, R4) (Figure 6A). This gave us a conservative map of eroGFP redox potential. As noted for cyroGFP, though the frequency distribution is quantitative in nature, the pixels in the different peaks cannot be resolved, as the peaks overlap. The thresholds are therefore somewhat arbitrary and hard; these therefore are only indicative of the nature of the redox potential. The color-based classification, in this case, is semi-quantitative at best, allowing us to comprehend the distribution of the redox potentials visually.

As expected, ER in most regions of the embryo was oxidizing in nature (Figure 6B,C). However, a transverse section of the 3D reconstructed images showed a layer with ER in a strongly reducing state just below the skin of the embryo. We checked the Ratio-2 distribution of eroGFP embryos and found that there were many pixels in the intermediate and strongly reducing states than expected from a normal distribution shown by the fitted black line (Figure 6D). Transverse section slices confirmed that the strongly reducing pixels (blue/green) were present as a layer surrounding the embryo, suggesting a layer of cells on or below the skin (Figure 6C). Small defined regions with pixels in the intermediate reducing state were present in the hindbrain and forebrain (Figure 6B,C (last two panels)).

These results suggested that there are regions in the embryo that harbor ER with lower redox potential than expected. However, roGFP2 is not a very sensitive probe for ER potential measurement because it is oxidized well below (−272 mV) the redox potential of ER (−180 mV to −235 mV). Thus, we chose the ERroGFPiE line to characterize differences in ER redox potential across different regions of the embryo.

**Figure 7:**
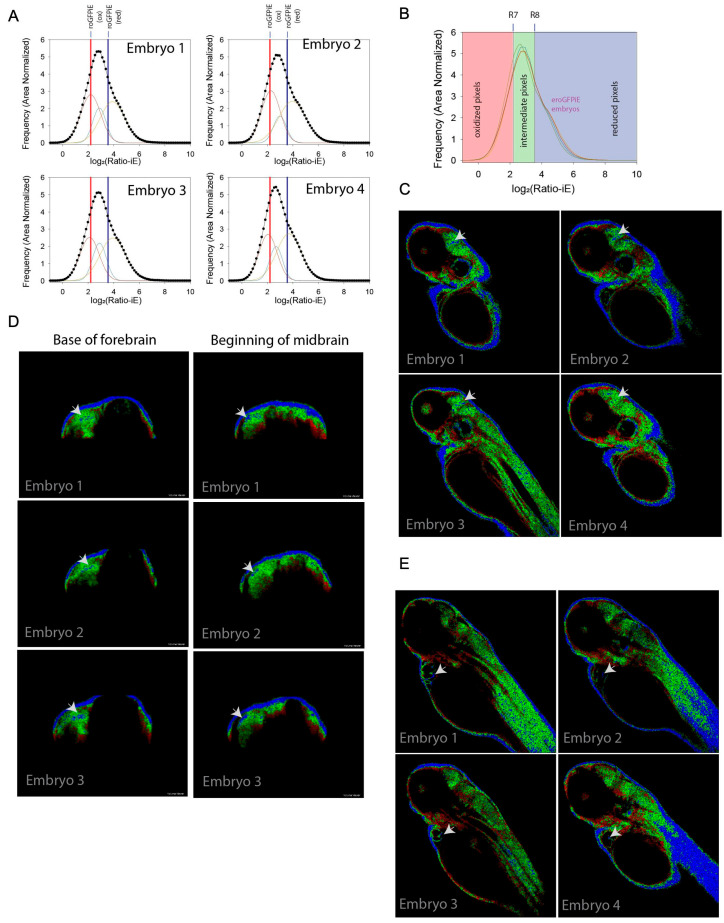
The more sensitive ERroGFPiE line reveals heterogeneity in the ER redox status across regions of the embryo. The 3 dpf live ERroGFPiE embryos were anesthetized, mounted in soft agar in lateral position, imaged, and analyzed for redox (Ratio-iE) values. The roGFPiE solutions treated with 10 mM DTT or 10 mM diamide were also imaged using the same settings, i.e., emission collection from 505 to 540 nm when first excited at 488 nm and then at 405 nm. (**A**) Frequency distribution for the log_2_ values of Ratio-iE for ERroGFPiE embryos were fitted to a combination of three normal distributions. The fitted peaks are shown by three smaller peaks, where red and blue line represents the Ratio-iE of fully oxidized (diamide treated) and reduced (DTT treated) roGFPiE solutions, respectively. Individual graphs were plotted for the 4 embryos. (**B**) The log_2_ Ratio-iE distribution for ERroGFPiE embryos was binned in three groups using reduced and oxidized Ratio-iE values from roGFPiE solutions. Ratios lower than the median of oxidized roGFPiE solution (R7 boundary) were oxidizing and given a red color. The values higher than the median of reduced roGFPiE solution (R8 boundary) were given a blue color (reducing regions) and intermediate values between R7 and R8 were given a green color. (**C**) Ratio-iE images of four roGFPiE embryos are shown using the described color scale where white arrow shows the reducing patches in the forebrain. (**D**) Ratio-iE images obtained after optical sectioning at forebrain (left panel) and midbrain (right panel) regions followed by rotation of the embryos to 0° are shown. White arrow indicates reducing patches in the base of forebrain and beginning of the midbrain in three independent embryos (top to bottom). (**E**) Ratio-iE images of the embryos in a different Z plane than C shows reduced status in the heart walls (white arrows) (Confocal microscope, 10× objective, scale bar 200 μm in **B**,**C**). Abbreviations: dpf—days post fertilization.

The ERroGFPiE probe indeed accentuates the deviations from expected in the redox potential of the ER across the embryo, as seen from the frequency distribution of Ratio-2 for ERroGFPiE (defined as Ratio-iE henceforth) (Figure 7A). Distribution of Ratio-iE did not fit well to a single-peak or a double-peak normal distribution and consists of at least three underlying normal distributions consistent among the different embryos tested (Figure 7A). The peak of the distribution of the lowest Ratio-iE corresponds well with the ratio obtained from completely oxidized roGFPiE, whereas the peak of the highest distribution corresponds well with the completely reduced roGFPiE (Figure 7A). Thus, roGFPiE does not suffer from the drawback of autofluorescence that shifted the ratio of eroGFP embryos towards lower values. This is primarily due to higher fluorescence of ERroGFPiE in the 405 nm channel, as also reported for roGFPiE variant [46]. Conservation of the distributions among different embryos also reinforces the observation that there are hyper-reducing regions in the embryos.

We defined the cutoffs for Ratio-iE based on the ratios obtained from measurements of the purified roGFPiE protein. Pixels having Ratio-iE more than the median Ratio-iE of reduced roGFPiE (DTT treated; R8 boundary) were marked as reducing regions (blue pixels), whereas pixels with Ratio-iE lower than that of oxidized roGFP-iE (diamide treated; R7 boundary) marked regions with oxidizing redox potential (red pixels) (Figure 7B). The mild deviations seen in the eroGFP embryos became clearer in ERroGFPiE as patches of reducing regions in the forebrain (blue pixels) (Figure 7C). From the transverse optical sections, we could see conserved regions of the reducing state at the caudal limits of the forebrain and the surface of the midbrain (Figure 7D). We also observed reducing regions possibly around the pericardium (Figure 7E). Images were taken at a higher magnification to confirm the locations of the reducing regions in the ERroGFPiE embryos (Figure S13A,B). At 40× magnification, we were able to observe similar striations of reducing regions in the forebrain (Figure S13B). The trunk region also had striations of reducing regions paralleling the chevron-shaped muscles (Figure S13B).

Our results suggest that the ER may not be as uniformly oxidizing throughout the embryo as previously thought (Figure 7A). The distribution of Ratio-iE in the embryos corroborates well with the heterogeneity seen in the redox potential of the ER. The lowest peak in the distribution coincides with completely reduced roGFPiE. The middle peak corresponds to a redox potential of ~−234 mV (Table 1) and is consistent between the different embryos. This peak corresponds roughly to the reported redox potential of the ER, as reported in some of the studies [55]. Roughly 26 ± 11% of the pixels represent the ER in this state; a similar percentage remains in a more oxidized (~22 ± 12%) state but most of the pixels represent the reduced (~51 ± 14%) state (Table 1). Minor interindividual variations are captured in the data along with some differences in experiments performed on different days (Table 1). However, the data largely remains consistent over days and across embryos, showing the robustness of the approach. These data, along with the images, suggest that there is not a single universal redox potential of the ER in the embryo. It is important to note that the pseudo-coloring is conservative and isolates only high-confidence hyper-oxidizing and hyper-reducing regions. Since the middle peak significantly overlaps with the low and high peaks, it is difficult to illustrate the range of heterogeneity using pseudo-colors. The heterogeneity in the redox potential may explain the range of redox values of the ER that have been measured in single-cell organisms or in cell lines [56,57,58,59]. Taken together, our study revealed that the redox state of cytosol and ER across the vertebrate embryos is not uniform and has many regions of heterogeneity.

### 3.6. ER Redox Potential Is Robustly Maintained When Proteostasis Is Challenged

**Figure 8:**
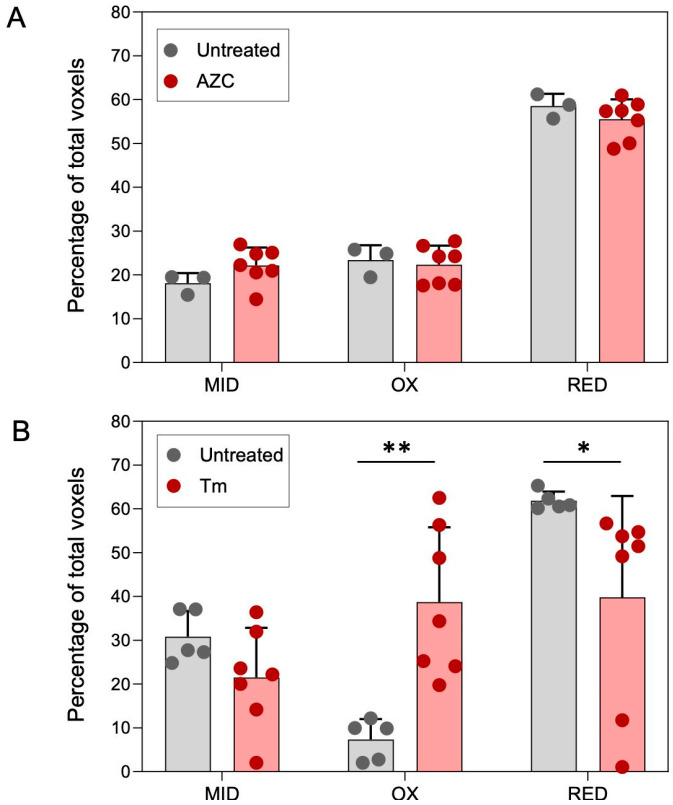
Redox state of ER is not perturbed upon proteotoxic challenges. Embryos were treated with 1 µg/mL Tm and 5 mM AZC at ~60 hpf and imaging was done after 12 h of treatment. Briefly, embryos were anesthetized, mounted in lateral position, and imaged sequentially, i.e., collection of emission signal first upon excitation at 488 nm, followed by excitation at 405 nm (confocal microscope, 10× objective). Analysis was done to calculate Ratio-iE values and percentage of voxels in each mid (intermediate), ox (oxidized), and red (reduced) bin was calculated after fitting the distributions to a 3-peak gaussian function, as shown in Figure 7A. Graph shows percentage of voxels in each bin plotted for AZC (**A**) and Tm (**B**) treated embryos with untreated embryos imaged on the same day. Each solid dot represents data from one embryo. Unpaired two tailed Student’s *t* test with unequal variance was performed to test significance, ** and * *p*-values are <0.01 and <0.05, respectively. (*n* = 3 and 5 for untreated in **A** and **B,** respectively, while *n* = 7 for both treatments; error bars show standard deviation). Abbreviations: hpf—hours post fertilization.

To check if the redox distribution is significantly perturbed when ER proteostasis or cellular proteostasis is perturbed, we treated the two dpf embryos with chemicals that alter ER proteostasis (Tunicamycin, Tm) or global cellular proteostasis (L-azetidine-2-carboxylic acid, AZC) and imaged them at 3 dpf. We also checked for the Hspa5 upregulation upon Tm treatment by Western blotting and we found a significant increase in Hspa5 compared to the WT control (Figure S14A,B). Similarly, we found Hsp90 upregulation upon AZC treatment, validating the effectiveness of the drug (Material not intended for publication [60]). Interestingly, AZC did not change the distribution or the mid-point redox potential of the ER (Table 2) (Figure 8A). By contrast, alteration of ER-proteostasis by Tm increased the embryo to embryo variability of the redox status (as seen from the large standard deviation) (Table 2) (Figure 8B). The treatment also altered the distribution, with a significant increase in the percentage of voxels in the oxidized (OX) bin when compared to untreated control embryos imaged on the same day. There was a significant decrease in reducing (RED) voxels too; however, we observed high biological variability as well (Table 2) (Figure 8B). The mid-point redox potential exhibited a slight decrease and shifted towards reducing potential, but this change was not significant either (Table 2). This indicates that the ER redox potential is robust and is maintained at constant values during challenges to proteostasis. However, the inter-individual differences are more during ER stress, indicating that some of the embryos were more affected by Tm induced ER stress than others. It will be important to understand the underlying basis of these interindividual differences during ER stress.

### 3.7. Limitations of the Study

While we base our conclusions on our findings, there are certain limitations to our study that need to be considered for future work in this area. We have assumed that the ER-targeting signal and the ER-retention signal are equally efficient in different cell types. There is currently no such report in zebrafish or any other vertebrate systems. Since Bip (GRP78/Hspa5) is a canonical ER chaperone in all tissues, we assumed the same to be true for zebrafish. In support of this assumption, we found that Hspa5 showed ER localization patterns in all the different cells we observed in our embryonic primary culture. Additionally, roGFP sensors are known to be pH-sensitive and may therefore report apparent changes in redox potential when organellar pH varies across different regions of the embryo. As this is the first study to generate an organism-wide map of ER redox status, there is currently no reference dataset for pH of ER distribution against which to compare our measurements. Consequently, some of the observed heterogeneity may or may not reflect differences in pH or other local ER environmental factors that influence sensor performance and could not be discerned.

## 4. Discussion

Sub-cellular milieu plays a very important role in facilitating or obstructing the catalytic reactions and biological processes that are executed in these compartments. Accordingly, each sub-cellular compartment maintains distinct pH, ionic concentrations and redox potential that are appropriate to the needs of the cell. The endoplasmic reticulum is the site of protein folding, disulphide bond formation, protein secretion, calcium storage, and lipid synthesis [61]. This necessitates specific conditions in the ER to facilitate these functions. For correct protein folding and disulphide formation, ER maintains an oxidizing environment. In contrast, cytosol is generally considered to be reducing. Previous studies, specifically in *C. elegans* (ER and cytosol both) and *Drosophila* (cytosol), have supported this view using in vivo redox sensors [27]. These and other studies in worms, *Arabidopsis thaliana*, and mice have shown that the redox potential of the cytosol is also responsive to different environmental conditions such as aging, time/light and disease [14,28,62,63,64,65]. However, it was not clear if, under physiological conditions, different tissues and organs would maintain a uniform redox potential in the ER; an organelle posited to maintain only an oxidizing redox potential physiologically. The zebrafish transgenic lines with ER-specific roGFP, eroGFP and ERroGFPiE allowed us to address this intriguing question. Our studies revealed that ER redox potential varies widely across the organism. Although generally considered oxidizing, our analysis showed regions in the animal where the ER was maintained at highly reducing conditions. The cytosol also showed a similar range of redox states, with some parts of the embryo having oxidizing conditions.

This is the first time that the redox potential of the ER has been compared across an individual vertebrate organism in non-pathological conditions. The differences we observed across tissue types in ER and cytosol redox conditions suggest that our longstanding assumption that ER is maintained in an oxidized state while cytosol is in a reducing state is simplistic. A large range of redox values has been reported in the literature. These were determined in cell culture models, and the measured values vary from −209 mV [59] to −231 ± 1.87 mV [57] to −240 mV (in tobacco leaves; [56]). Within a vertebrate organism, we reconcile this diversity and show that cells from different regions of the embryo maintain their ER in different redox states. We believe this happens as different tissues within an organism and different cells within a tissue fulfill different biological roles. For instance, the ER in pancreatic alpha cells would primarily be folding and secreting proteins while the ER in the muscle would primarily be acting as calcium stores. This would warrant different redox milieu within these organelles in the pancreas and muscle. Our study finds very striking differences in the ER and the cytosol redox state in select regions of the brain, suggesting interesting differences in cellular processes within different regions of the brain.

## 5. Innovation

This study provides the real-time redox state in the ER of a vertebrate model system, and we have observed regions of redox anomaly. The full dataset in raw format is hosted on the Dataverse server (https://doi.org/10.7910/DVN/PRCFJM) and we hope that researchers are able to identify their favorite regions from the dataset for further analysis. We have also developed a data analysis pipeline to remove yolk autofluorescence from zebrafish embryos, which was a major limitation earlier in such studies.

## Figures and Tables

**Table 1:** Properties of the different peaks of Ratio-iE (shown as a percentage of voxels) and redox potential, as obtained from ERroGFPiE embryos.

		Percentage of Voxels in Each Redox Bin			Redox Potential
Day	Embryo Tag	MID	OX	RED	Middle Peak (mV)
Day 1	embryo 1	19.15	39.30	41.55	−230.50
	embryo 2	13.95	39.88	46.17	−231.96
	embryo 3	21.10	33.97	44.93	−232.15
	embryo 4	18.11	34.22	47.66	−229.99
Day 2	embryo 1	24.82	9.88	65.30	−237.03
	embryo 2	37.08	2.78	60.14	−234.09
	embryo 3	27.31	12.16	60.54	−234.21
	embryo 4	27.74	9.95	62.31	−234.86
	embryo 5	37.10	2.02	60.87	−234.34
Day 3	embryo 1	19.35	19.44	61.21	−237.58
	embryo 2	15.43	25.78	58.79	−236.32
	embryo 3	19.49	24.85	55.66	−235.47
Day 4	embryo 1	28.05	18.54	53.41	−235.65
	embryo 2 ^#^	61.74	34.57	3.69	−228.45
	embryo 3	23.10	25.08	51.82	−236.47
	embryo 4	26.84	28.54	44.63	−236.22
AVERAGE		26.27	22.56	51.17	−234.08
SD		11.23	12.03	14.22	2.63

^#^ This embryo exhibits an outlier distribution. **Column Legend**—DAY: Experiments done on different days are labeled as separate sets. EMBRYO TAG: Each embryo done on a day is serially labeled. OX: Percentage of voxels exhibiting oxidizing redox potential. RED: Percentage of voxels exhibiting reducing redox potential. MID: Percentage of voxels exhibiting intermediate redox potential. MIDDLE PEAK (mV): Calculated redox potential of the intermediate peak (MID) in millivolts. SD: Standard deviation.

**Table 2:** Properties of Ratio-iE and redox potential values as obtained from ERroGFPiE embryos when treated with AZC and Tm.

		Percentage of Voxels in Each Redox Bin			Redox Potential
Treatment	Embryo Tag	MID	OX	RED	Middle Peak (mV)
AZC	embryo 1	24.82	17.75	57.43	−236.33
	embryo 2	26.95	24.25	48.80	−237.17
	embryo 3	20.92	18.12	60.96	−236.94
	embryo 4	20.53	24.19	55.29	−235.06
	embryo 5	25.08	17.57	57.35	−235.17
	embryo 6	14.46	26.64	58.90	−236.68
	embryo 7	22.26	27.68	50.06	−235.23
	AVERAGE	22.14	22.31	55.54	−236.08
	SD	3.82	4.06	4.19	0.84
Tunicamycin	embryo 1	31.96	56.30	11.75	−237.14
	embryo 2	2.02	48.80	49.18	−267.89
	embryo 3	23.58	19.77	56.65	−234.67
	embryo 4	36.42	62.52	1.07	−264.06
	embryo 5	20.03	25.26	54.71	−233.41
	embryo 6	14.20	34.34	51.46	−234.35
	embryo 7	22.19	24.07	53.74	−235.12
	AVERAGE	21.49	38.72	39.79	−243.81
	SD	10.50	15.82	21.42	14.10

**Column legend:** TREATMENT: Experiments labeled with the chemicals used to perturb proteostasis. Either AZC (azetidine-2-carboxylic acid) at 5 mM or Tunicamycin at 1 μg/mL were used to treat the embryos for 12 h. EMBRYO TAG: Each embryo done on a day is serially labeled. OX: Percentage of voxels exhibiting oxidizing redox potential. RED: Percentage of voxels exhibiting reducing redox potential. MID: Percentage of voxels exhibiting intermediate redox potential. MIDDLE PEAK (mV): Calculated redox potential of the intermediate peak (MID) in millivolts. SD: Standard deviation.

## Data Availability

The original data of the Figures generated in this study are openly available on Dataverse (https://dataverse.harvard.edu/dataset.xhtml?persistentId=doi:10.7910/DVN/PRCFJM; accessed on 6 December 2021) and Zenodo (https://zenodo.org/records/19600314; DOI: 10.5281/zenodo.19600313; accessed on 24 April 2026).

## Supplementary Materials

The following supporting information can be downloaded at https://www.mdpi.com/article/10.3390/biomedicines14071585/s1: Figure S1: roGFP is expressed ubiquitously in all transgenic lines. Figure S2: Signal peptide cleavage prediction revealed proper cleavage of both the signal sequences used. Figure S3: roGFP probes are localized in the desired organelles in generated transgenic lines. Figure S4: A-H Representative images for ER Tracker Red colocalization with roGFP2 in eroGFP line. Figure S5: roGFP transgenic lines for ER do not exhibit perturbations in homeostasis. Figure S6: roGFP transgenic lines show redox sensitive properties in vivo. Figure S7: Ratio-2 distribution of primary cell culture from 1 dpf zebrafish embryos. Figure S8: The histogram of ratios (A), or the median ratio of the frequency distribution (B) computed from the full stack of the all the images are plotted. Figure S9: A representative Figure to show that the ratio is effectively calculated from the non-autofluorescent regions of the zebrafish larvae. Figure S10: Confocal imaging enables to procure signals from either half of the embryo in 3D. Figure S11: Ratio-2 distribution of eroGFP and cyroGFP fish lines showed shift in the peak as compared to the oxidized and reduced roGFP2 solution. Figure S12: Bright field images of embryos shown for redox imaging. Figure S13: Variations in redox ratios of ER are more apparent with higher magnification images of ERroGFPiE embryos. Figure S14: Tm treatment induced misfolding stress revealed by upregulation of Hspa5. Table S1: Primers used for the generation of transgenic lines.

## Abbreviations

The abbreviations used in this study are: ER, endoplasmic reticulum; PDI, protein disulfide isomerase; GSH, glutathione reduced; GSSG, glutathione oxidized; dpf, days post fertilization; ss, signal sequence; a.a., amino acids; DTT, dithiothreitol; Tm, tunicamycin; ROS, reactive oxygen species; and diamide, N,N,N′,N′-tetramethylazodicarboxamide.

## References

[1] Ellgaard L., Helenius A. (2003). Quality control in the endoplasmic reticulum. Nat. Rev. Mol. Cell Biol..

[2] Ellgaard L., Ruddock L.W. (2005). The human protein disulphide isomerase family: Substrate interactions and functional properties. EMBO Rep..

[3] Gutscher M., Pauleau A.L., Marty L., Brach T., Wabnitz G.H., Samstag Y., Meyer A.J., Dick T.P. (2008). Real-time imaging of the intracellular glutathione redox potential. Nat. Methods.

[4] Hanson G.T., Aggeler R., Oglesbee D., Cannon M., Capaldi R.A., Tsien R.Y., Remington S.J. (2004). Investigating mitochondrial redox potential with redox-sensitive green fluorescent protein indicators. J. Biol. Chem..

[5] Hwang C., Sinskey A.J., Lodish H.F. (1992). Oxidized redox state of glutathione in the endoplasmic reticulum. Science.

[6] Østergaard H., Tachibana C., Winther J.R. (2004). Monitoring disulfide bond formation in the eukaryotic cytosol. J. Cell Biol..

[7] Montero D., Tachibana C., Rahr Winther J., Appenzeller-Herzog C. (2013). Intracellular glutathione pools are heterogeneously concentrated. Redox Biol..

[8] Morgan B., Ezeriņa D., Amoako T.N., Riemer J., Seedorf M., Dick T.P. (2013). Multiple glutathione disulfide removal pathways mediate cytosolic redox homeostasis. Nat. Chem. Biol..

[9] Ayer A., Sanwald J., Pillay B.A., Meyer A.J., Perrone G.G., Dawes I.W. (2013). Distinct redox regulation in sub-cellular compartments in response to various stress conditions in Saccharomyces cerevisiae. PLoS ONE.

[10] Birk J., Meyer M., Aller I., Hansen H.G., Odermatt A., Dick T.P., Meyer A.J., Appenzeller-Herzog C. (2013). Endoplasmic reticulum: Reduced and oxidized glutathione revisited. J. Cell Sci..

[11] Hu J., Dong L., Outten C.E. (2008). The redox environment in the mitochondrial intermembrane space is maintained separately from the cytosol and matrix. J. Biol. Chem..

[12] Romero-Aristizabal C., Marks D.S., Fontana W., Apfeld J. (2014). Regulated spatial organization and sensitivity of cytosolic protein oxidation in Caenorhabditis elegans. Nat. Commun..

[13] Adhish M., Manjubala I. (2023). Effectiveness of zebrafish models in understanding human diseases—A review of models. Heliyon.

[14] Chen Y., Zhi L., Cui S., Wang H., Zeng C., Zhang H. (2025). The Human LRRK2-R1441G Mutation Drives Age-Dependent Oxidative Stress and Mitochondrial Dysfunction in Dopaminergic Neurons. Res. Sq..

[15] Pöschel S., Müller M. (2026). Quantitative imaging of mitochondrial redox conditions at the single-organelle level. Mitochondrion.

[16] Inui M., Saito A., Fleischer S. (1987). Purification of the ryanodine receptor and identity with feet structures of junctional terminal cisternae of sarcoplasmic reticulum from fast skeletal muscle. J. Biol. Chem..

[17] Lai F.A., Erickson H.P., Rousseau E., Liu Q.Y., Meissner G. (1988). Purification and reconstitution of the calcium release channel from skeletal muscle. Nature.

[18] Glaumann H., Bergstrand A., Ericsson J.L. (1975). Studies on the synthesis and intracellular transport of lipoprotein particles in rat liver. J. Cell Biol..

[19] Vidugiriene J., Menon A.K. (1993). Early lipid intermediates in glycosyl-phosphatidylinositol anchor assembly are synthesized in the ER and located in the cytoplasmic leaflet of the ER membrane bilayer. J. Cell Biol..

[20] Baumann O., Walz B. (2001). Endoplasmic reticulum of animal cells and its organization into structural and functional domains. Int. Rev. Cytol..

[21] Sitia R., Meldolesi J. (1992). Endoplasmic reticulum: A dynamic patchwork of specialized subregions. Mol. Biol. Cell.

[22] Zheng H.Q., Staehelin L.A. (2001). Nodal endoplasmic reticulum, a specialized form of endoplasmic reticulum found in gravity-sensing root tip columella cells. Plant Physiol..

[23] Ghaemmaghami S., Huh W.K., Bower K., Howson R.W., Belle A., Dephoure N., O’Shea E.K., Weissman J.S. (2003). Global analysis of protein expression in yeast. Nature.

[24] Burdakov D., Petersen O.H., Verkhratsky A. (2005). Intraluminal calcium as a primary regulator of endoplasmic reticulum function. Cell Calcium.

[25] Takeshima H. (2002). Intracellular Ca2+ store in embryonic cardiac myocytes. Front. Biosci. A J. Virtual Libr..

[26] Coussin F., Macrez N., Morel J.L., Mironneau J. (2000). Requirement of ryanodine receptor subtypes 1 and 2 for Ca^2+^-induced Ca^2+^ release in vascular myocytes. J. Biol. Chem..

[27] Albrecht S.C., Barata A.G., Grosshans J., Teleman A.A., Dick T.P. (2011). In vivo mapping of hydrogen peroxide and oxidized glutathione reveals chemical and regional specificity of redox homeostasis. Cell Metab..

[28] Kirstein J., Morito D., Kakihana T., Sugihara M., Minnen A., Hipp M.S., Nussbaum-Krammer C., Kasturi P., Hartl F.U., Nagata K. (2015). Proteotoxic stress and ageing triggers the loss of redox homeostasis across cellular compartments. EMBO J..

[29] Knoke L.R., Zimmermann J., Lupilov N., Schneider J.F., Celebi B., Morgan B., Leichert L.I. (2023). The role of glutathione in periplasmic redox homeostasis and oxidative protein folding in Escherichia coli. Redox Biol..

[30] Chandrasekharan A., Varadarajan S.N., Lekshmi A., Santhoshkumar T. (2023). Real-time simultaneous imaging of temporal alterations in cytoplasmic and mitochondrial redox in single cells during cell division and cell death. Free Radic. Biol. Med..

[31] Jain Tiffee P.J., Sivasailam A., Kumar K.S., Varghese Jancy S., Geetha Jayaprasad A., Halikar A.M., Rather A.A., Satheesan Sinivirgin N., Anurup K.G., Santhoshkumar T.R. (2026). Time-resolved simultaneous imaging of mitochondrial reactive oxygen species and lysosomal permeabilization to determine organelle-centred cell death. Redox Rep..

[32] Davies B.M., Katayama J.K., Monsivais J.E., Adams J.R., Dilts M.E., Eberting A.L., Hansen J.M. (2023). Real-time analysis of dynamic compartmentalized GSH redox shifts and H2O2 availability in undifferentiated and differentiated cells. Biochim. Biophys. Acta (BBA)-Gen. Subj..

[33] Teixeira R.B., Pfeiffer M., Zhang P., Shafique E., Rayta B., Karbasiafshar C., Ahsan N., Sellke F.W., Abid M.R. (2023). Reduction in mitochondrial ROS improves oxidative phosphorylation and provides resilience to coronary endothelium in non-reperfused myocardial infarction. Basic Res. Cardiol..

[34] O’Donnell K.C., Vargas M.E., Sagasti A. (2013). WldS and PGC-1α regulate mitochondrial transport and oxidation state after axonal injury. J. Neurosci..

[35] Pak V.V., Ezeriņa D., Lyublinskaya O.G., Pedre B., Tyurin-Kuzmin P.A., Mishina N.M., Thauvin M., Young D., Wahni K., Martínez Gache S.A. (2020). Ultrasensitive Genetically Encoded Indicator for Hydrogen Peroxide Identifies Roles for the Oxidant in Cell Migration and Mitochondrial Function. Cell Metab..

[36] Panieri E., Millia C., Santoro M.M. (2017). Real-time quantification of subcellular H(2)O(2) and glutathione redox potential in living cardiovascular tissues. Free Radic. Biol. Med..

[37] Hamre K., Zhang W., Austgulen M.H., Mykkeltvedt E., Yin P., Berntssen M., Espe M., Berndt C. (2024). Systemic and strict regulation of the glutathione redox state in mitochondria and cytosol is needed for zebrafish ontogeny. Biochim. Biophys. Acta Gen. Subj..

[38] Westerfield M. (1994). The Zebrafish Book: A Guide for the Laboratory Use of Zebrafish (Brachydanio Rerio).

[39] Zámbó B., Bartos Z., Mózner O., Szabó E., Várady G., Poór G., Pálinkás M., Andrikovics H., Hegedűs T., Homolya L. (2018). Clinically relevant mutations in the ABCG2 transporter uncovered by genetic analysis linked to erythrocyte membrane protein expression. Sci. Rep..

[40] Starck S.R., Tsai J.C., Chen K., Shodiya M., Wang L., Yahiro K., Martins-Green M., Shastri N., Walter P. (2016). Translation from the 5′ untranslated region shapes the integrated stress response. Science.

[41] Leder A., Mas G., Szentgyörgyi V., Jakob R.P., Maier T., Spang A., Hiller S. (2025). A multichaperone condensate enhances protein folding in the endoplasmic reticulum. Nat. Cell Biol..

[42] Kim J.H., Seong S., Kim K., Kim I., Koh J.T., Kim N. (2026). Molecular role of developmentally regulated GTP-binding protein 1 in coordinating osteoclast and osteoblast differentiation during bone remodeling. Mol. Cells.

[43] Jassey A., Paudel B., Wagner M.A., Pollack N., Cheng I.T., Godoy-Ruiz R., Weber D.J., Jackson W.T. (2026). Enterovirus-induced cleavage of Mitofusin 2 generates mitophagosomes for enveloped virion release. Sci. Adv..

[44] Shanmugam G., Narasimhan M., Tamowski S., Darley-Usmar V., Rajasekaran N.S. (2017). Constitutive activation of Nrf2 induces a stable reductive state in the mouse myocardium. Redox Biol..

[45] Bhaskar A., Chawla M., Mehta M., Parikh P., Chandra P., Bhave D., Kumar D., Carroll K.S., Singh A. (2014). Reengineering redox sensitive GFP to measure mycothiol redox potential of Mycobacterium tuberculosis during infection. PLoS Pathog..

[46] Lohman J.R., Remington S.J. (2008). Development of a family of redox-sensitive green fluorescent protein indicators for use in relatively oxidizing subcellular environments. Biochemistry.

[47] Merksamer P.I., Trusina A., Papa F.R. (2008). Real-time redox measurements during endoplasmic reticulum stress reveal interlinked protein folding functions. Cell.

[48] Kanekura K., Ishigaki S., Merksamer P.I., Papa F.R., Urano F. (2013). Establishment of a system for monitoring endoplasmic reticulum redox state in mammalian cells. Lab. Investig. A J. Tech. Methods Pathol..

[49] Kozutsumi Y., Segal M., Normington K., Gething M.J., Sambrook J. (1988). The presence of malfolded proteins in the endoplasmic reticulum signals the induction of glucose-regulated proteins. Nature.

[50] Kasahara E., Lin L.R., Ho Y.S., Reddy V.N. (2005). SOD2 protects against oxidation-induced apoptosis in mouse retinal pigment epithelium: Implications for age-related macular degeneration. Investig. Ophthalmol. Vis. Sci..

[51] Wang Y., Branicky R., Noë A., Hekimi S. (2018). Superoxide dismutases: Dual roles in controlling ROS damage and regulating ROS signaling. J. Cell Biol..

[52] Harding H.P., Zhang Y., Zeng H., Novoa I., Lu P.D., Calfon M., Sadri N., Yun C., Popko B., Paules R. (2003). An integrated stress response regulates amino acid metabolism and resistance to oxidative stress. Mol. Cell.

[53] Zeeshan H.M., Lee G.H., Kim H.R., Chae H.J. (2016). Endoplasmic Reticulum Stress and Associated ROS. Int. J. Mol. Sci..

[54] Igbaria A., Merksamer P.I., Trusina A., Tilahun F., Johnson J.R., Brandman O., Krogan N.J., Weissman J.S., Papa F.R. (2019). Chaperone-mediated reflux of secretory proteins to the cytosol during endoplasmic reticulum stress. Proc. Natl. Acad. Sci. USA.

[55] Delic M., Mattanovich D., Gasser B. (2010). Monitoring intracellular redox conditions in the endoplasmic reticulum of living yeasts. FEMS Microbiol. Lett..

[56] Brach T., Soyk S., Müller C., Hinz G., Hell R., Brandizzi F., Meyer A.J. (2009). Non-invasive topology analysis of membrane proteins in the secretory pathway. Plant J. Cell Mol. Biol..

[57] van Lith M., Tiwari S., Pediani J., Milligan G., Bulleid N.J. (2011). Real-time monitoring of redox changes in the mammalian endoplasmic reticulum. J. Cell Sci..

[58] Schuiki I., Zhang L., Volchuk A. (2012). Endoplasmic reticulum redox state is not perturbed by pharmacological or pathological endoplasmic reticulum stress in live pancreatic β-cells. PLoS ONE.

[59] Avezov E., Cross B.C., Kaminski Schierle G.S., Winters M., Harding H.P., Melo E.P., Kaminski C.F., Ron D. (2013). Lifetime imaging of a fluorescent protein sensor reveals surprising stability of ER thiol redox. J. Cell Biol..

[60] Verma M., Sachidanandan C., Chakraborty K. (2022). AZC treatment in zebrafish embryos induces Hsp90.

[61] Schwarz D.S., Blower M.D. (2016). The endoplasmic reticulum: Structure, function and response to cellular signaling. Cell. Mol. Life Sci. CMLS.

[62] Meyer A.J., Brach T., Marty L., Kreye S., Rouhier N., Jacquot J.P., Hell R. (2007). Redox-sensitive GFP in Arabidopsis thaliana is a quantitative biosensor for the redox potential of the cellular glutathione redox buffer. Plant J. Cell Mol. Biol..

[63] Festerling K., Can K., Kügler S., Müller M. (2020). Overshooting Subcellular Redox-Responses in Rett-Mouse Hippocampus during Neurotransmitter Stimulation. Cells.

[64] Collado-Arenal A.M., Exposito-Rodriguez M., Mullineaux P.M., Olmedilla A., Romero-Puertas M.C., Sandalio L.M. (2024). Cadmium exposure induced light/dark- and time-dependent redox changes at subcellular level in Arabidopsis plants. J. Hazard. Mater..

[65] Woeste H., van Agen L., Müller M. (2025). Mapping of neuronal redox conditions in a mouse model of Rett syndrome. Neuroimage Rep..

