# Supplementary material for: Live Visualization of Endoplasmic Reticulum Redox Potential in Zebrafish Embryos Reveals Region-Specific Heterogeneity
Source: Biomedicines. 2026 Jul 15;14(7):1585. doi: 10.3390/biomedicines14071585 (PMC13405980; doi:10.3390/biomedicines14071585)
Supplement: Supplementary file 1 [file biomedicines-14-01585-s001.zip › biomedicines-4292327-supplementary.pdf]

# Supplemental Information

# Live visualization of endoplasmic reticulum redox potential in zebrafish embryos reveals region-specific heterogeneity

Monika Verma, Niraj Rajesh Bhatt, Koushika Chandrasekaran, Aseem Chaphalkar, Kriti Verma, Shreyansh Umale, Shweta Verma, Chetana Sachidanandan, and Kausik Chakraborty

## Inventory of supplemental items

1. Figure S1 - independent
2. Figure S2 - independent
3. Figure S3 - relates to main Figure 1
4. Figure S4 - relates to main Figure 1
5. Figure S5 - independent
6. Figure S6 - independent
7. Figure S7 - relates to main Figure 2
8. Figure S8 - relates to main Figure 3
9. Figure S9 - relates to main Figure 3
10. Figure S10 - relates to main Figure 4
11. Figure S11 - relates to main Figure 5 and 6
12. Figure S12 - relates to main Figure 5, 6, and 7
13. Figure S13 - relates to main Figure 7
13. Figure S14 – relates to main Figure 8
14. Table S1- independent

Supplementary figure S1

A

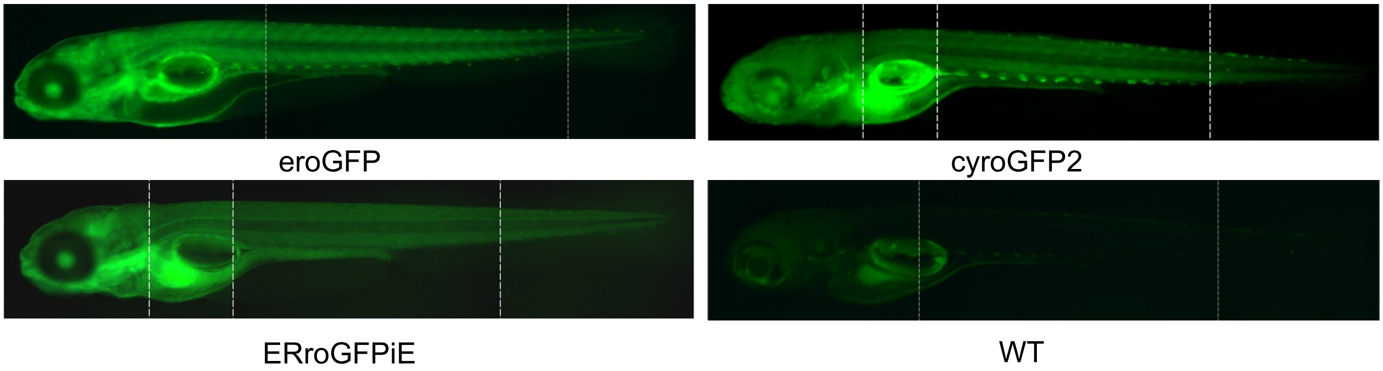


**Supplementary figure S1: roGFP is expressed ubiquitously in all transgenic lines.** (A) 4 dpf embryos were anaesthetized and mounted in lateral position in 2.5% methyl cellulose. Representative fluorescence images of the three transgenic lines and wild type is shown (epifluorescence microscope, 5X objective). White dotted lines show stitching of multiple images together to display the embryos completely. Abbreviations- dpf= days post fertilization.

Supplementary figure S2

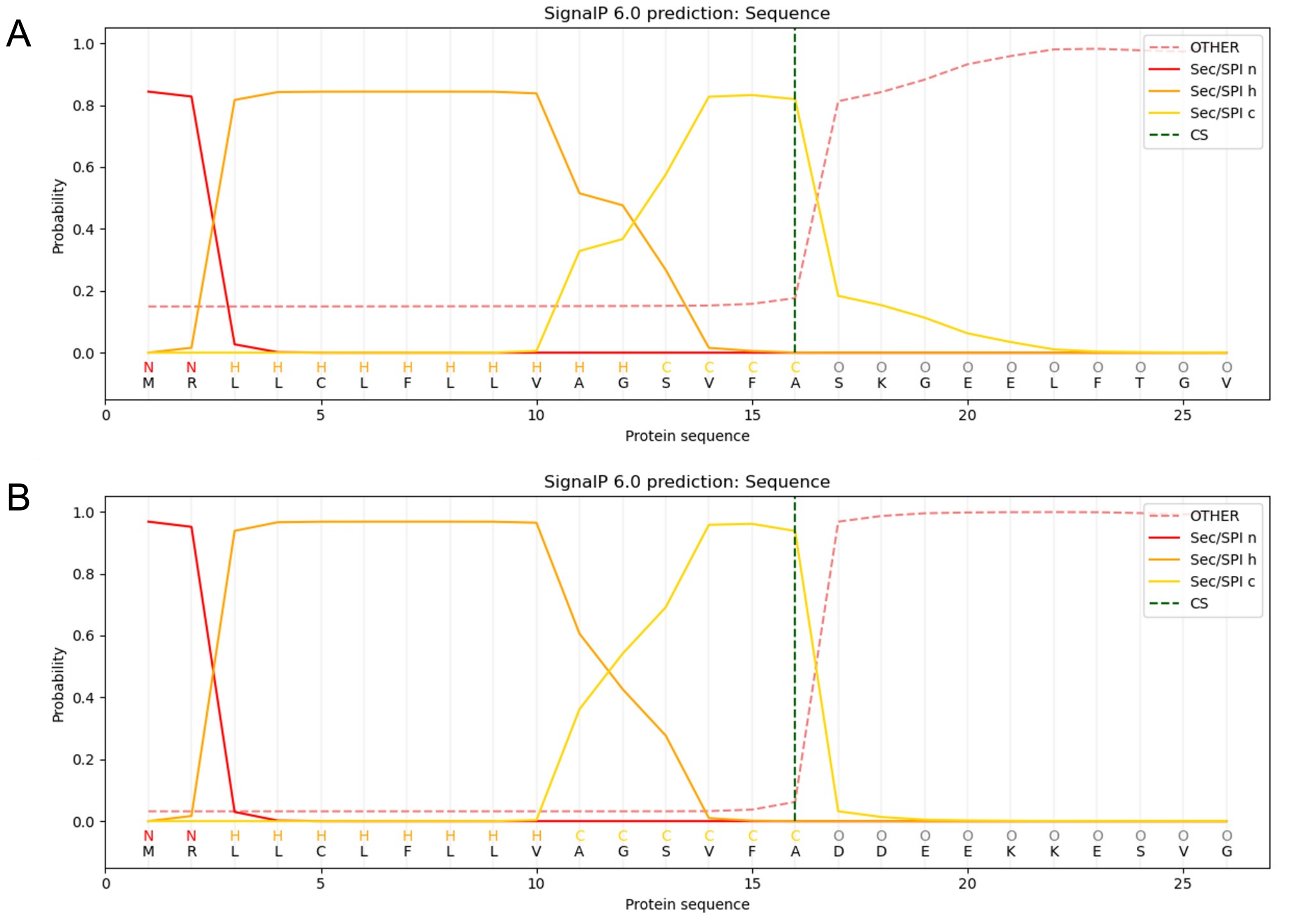


**Supplementary figure S 2: Signal peptide cleavage prediction revealed proper cleavage of both the signal sequences used.** We used free online software, SignalP 6.0, to predict signal peptide cleavage site for the signal sequences we used for roGFP transgenic lines. (A) First 16 a.a. of Hspa5 (GRP78) followed by first 10 a.a. (after removing methionine) of roGFP2 were used for the prediction. (B) First 26 a.a. of Hspa5 (GRP78) were used for prediction. The black dotted line is the predicted residue after which cleavage would be done. Abbreviations- a.a.- amino acid, CS- cleavage site, Sec/SPI- transported by Sec translocon and cleaved by Signal Peptidase I, n h and c are N-terminal, hydrophobic and C-terminal regions of signal peptide.

Supplementary figure S3

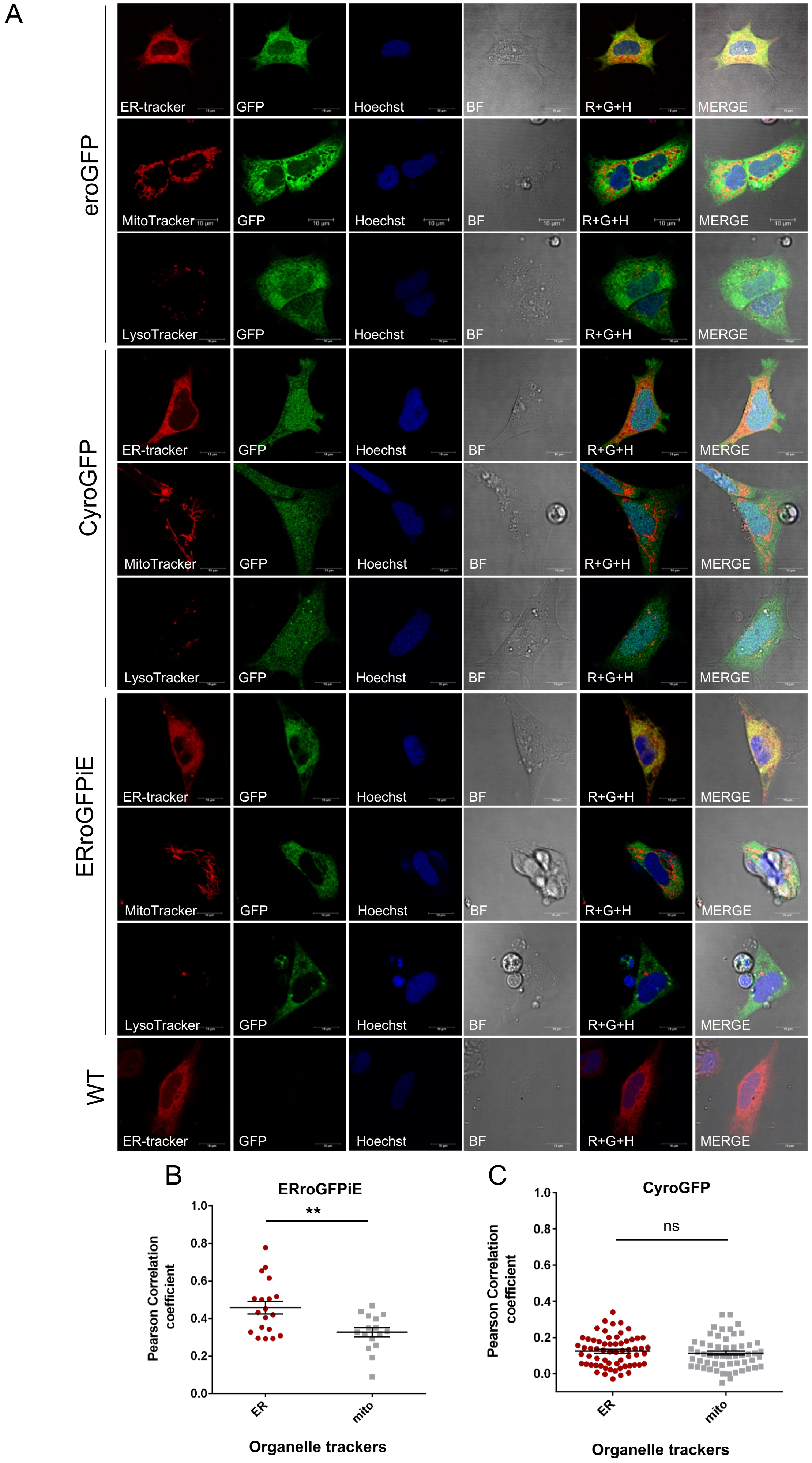

**Supplementary figure S3: roGFP probes are localized in the desired organelles in generated transgenic lines.** (A) Single cell suspension was made form 1 dpf embryos and cells were attached to substratum (8 chambered coverglasses) in L15 medium at 28°C. Imaging was done after overnight attachment of live cells with different organelle stainings. Fluorescence image of zebrafish primary culture cells with ER, Mito and LysoTracker staining for all the transgenic lines are shown, with wild type fish as a negative control. These panels correspond to the images of transgenic lines as labeled. The first image in each panel is ER, Mito or LysoTracker followed by the GFP channel corresponding to roGFP. Third image is Hoechst staining for nucleus and next image is bright field (confocal microscope, 63X objective, 4X zoom, scale 10 µm). (B) and (C) Scatter plot shows Pearson correlation coefficient of green and red channel for ERroGFPiE and cyroGFP line respectively, where each dot is one cell (primary cells derived from n= 100 embryos per replicate; represents data for 3 biological replicates). Two tailed Student's t test with unequal variance was done to test significance, ** is p value <0.01. Abbreviations- dpf= days post fertilization, BF= bright field, R+G+H= merge of red, green and Hoechst channel, ER= endoplasmic reticulum, mito= mitochondria.

Supplementary figure S4

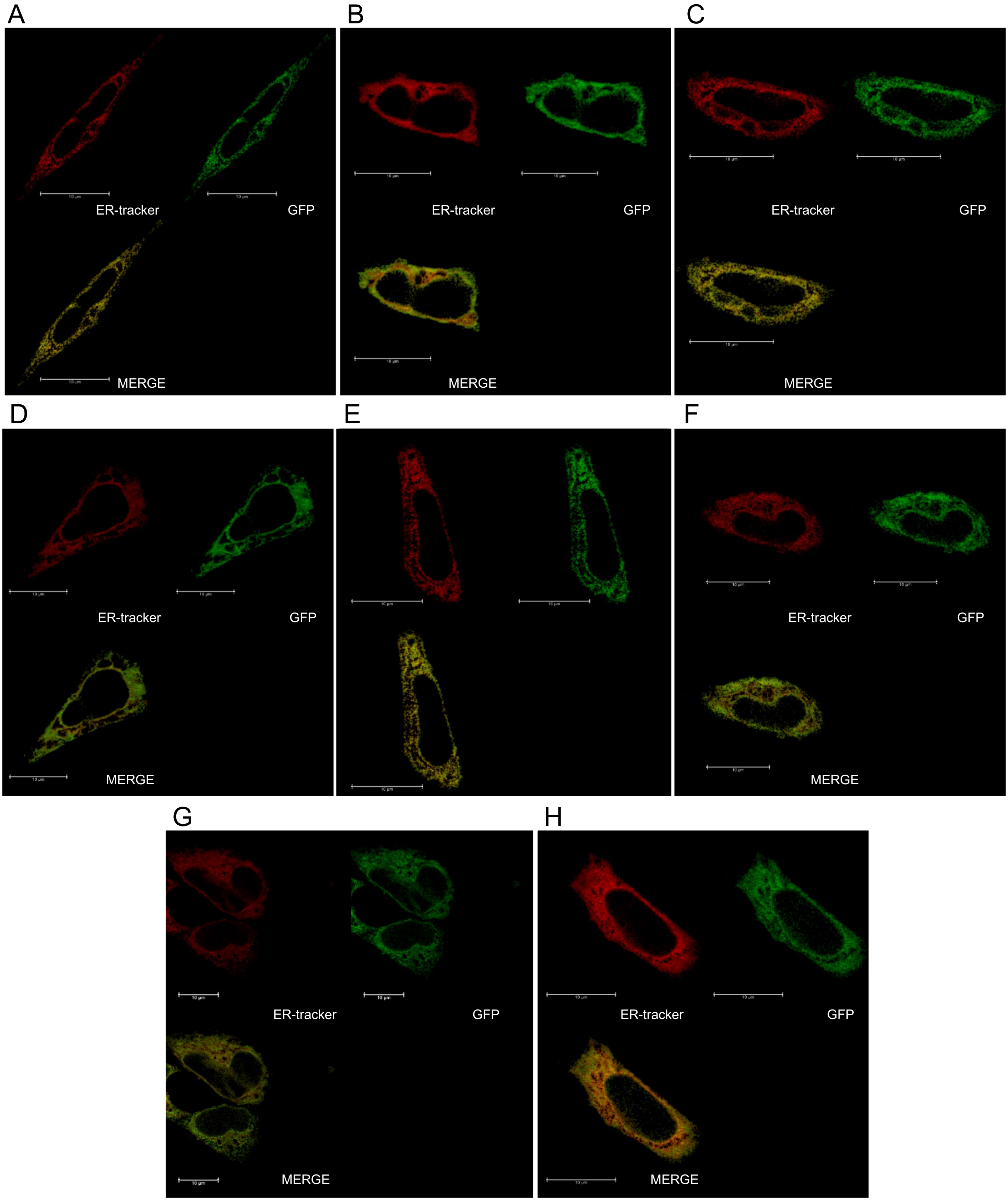


**Supplementary figure S4: A-H Representative images for ER Tracker Red colocalization with roGFP2 in eroGFP line.** Fluorescence images of zebrafish primary culture cells with ER Tracker staining for eroGFP line are shown. The first image in upper panel is ER Tracker Red, followed by the GFP channel corresponding to roGFP2. Image in lower panel image is merge of red and green channel showing yellow pixels (confocal microscope, 63X objective, 4X zoom, scale 10µm).

Supplementary figure S5

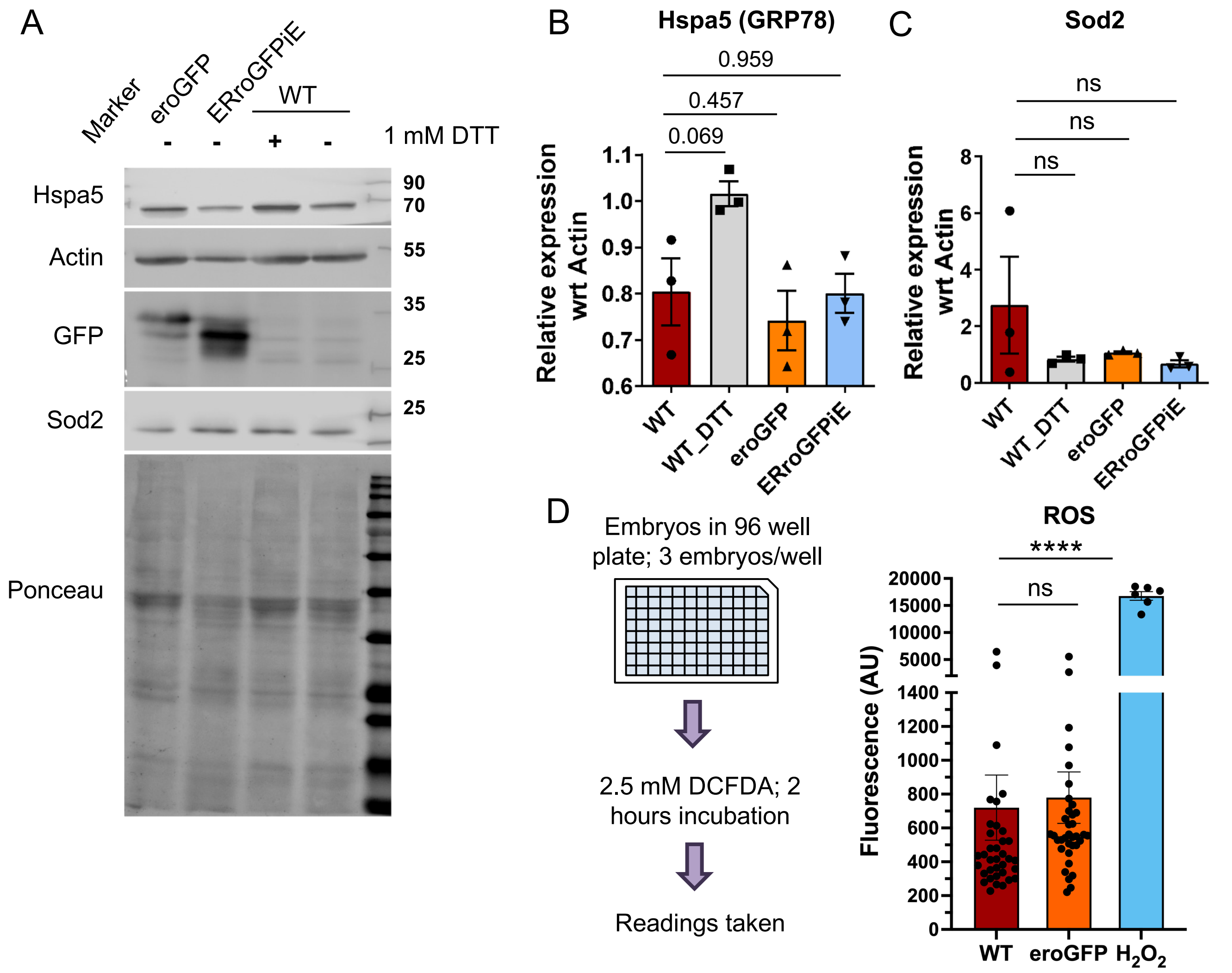


**Supplementary figure S5: roGFP transgenic lines for ER do not exhibit perturbations in homeostasis.** (A) Protein lysates from 5 dpf embryos (n=39) were loaded on a SDS gel and the transfer was done to membrane. Western blots show expression of Hspa5 (GRP78) and Sod2 in the ER transgenic lines and wild type along with 1mM DTT treated embryos (8 h) taken as a positive control. GFP was also probed, and actin was used as a loading control. (B) and (C) The graph shows quantification of Hspa5 and Sod2 expression w.r.t. actin respectively which was done using ImageJ. Paired Student's t-Test with two tails was performed using Microsoft excel. The bars show mean while error bars show SEM. (D) 3 live embryos at 5 dpf were put in each well of a 96 well plate. Water was removed to an extent that embryos have some water to survive and DCFDA dye was added. Incubation was done for 2 h and readings were taken. Wells with embryos incubated in $H_2O_2$ was used as a positive control. 12 wells were used for one replicate, so each replicate had 36 embryos, and each dot represents reading from one well. Unpaired Student's t-Test was done with Welch correction using Graph pad Prism. Bars represents mean from 3 replicates while error bars are SEM. <0.0001Abbreviations- dpf= days post fertilization, WT= wild type, w.r.t= with respect to, SEM= standard error around mean.

Supplementary figure S6

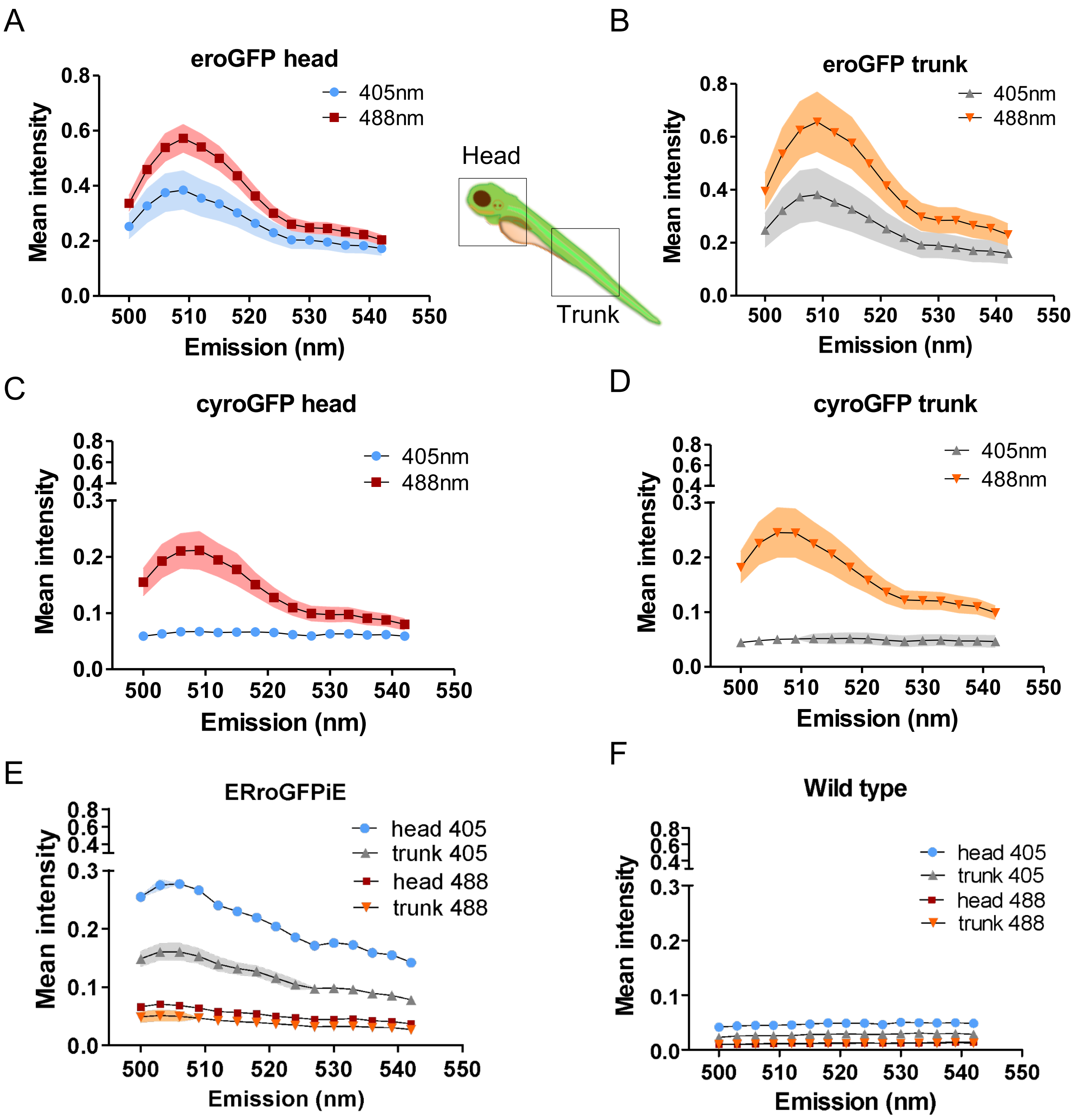


**Supplementary figure S6: roGFP transgenic lines show redox sensitive properties *in vivo.*** Embryos were excited at first 405 nm, and emission scan was taken from 500 nm to 540 nm, then excited at 488 nm and emission was collected in the same range. These graphs show emission scan for live embryos at 4 dpf where head and trunk regions are marked in the cartoon representation of an embryo. (A) and (B) Graphs show emission peaks when excited at 405 and 488 nm for head and trunk regions of eroGFP line, respectively. (C), and (D) Graphs show emission peaks at 405 and 488 nm for head and trunk regions of cyroGFP line respectively (E) and (F) Peaks at 405 and 488 nm for ERroGFPiE and WT lines are shown respectively, settings were kept same for all. Each line represents mean value from 4 replicates and shaded region indicates SEM. Abbreviations- dpf= days post fertilization, SEM= standard error around mean.

Supplementary figure S7

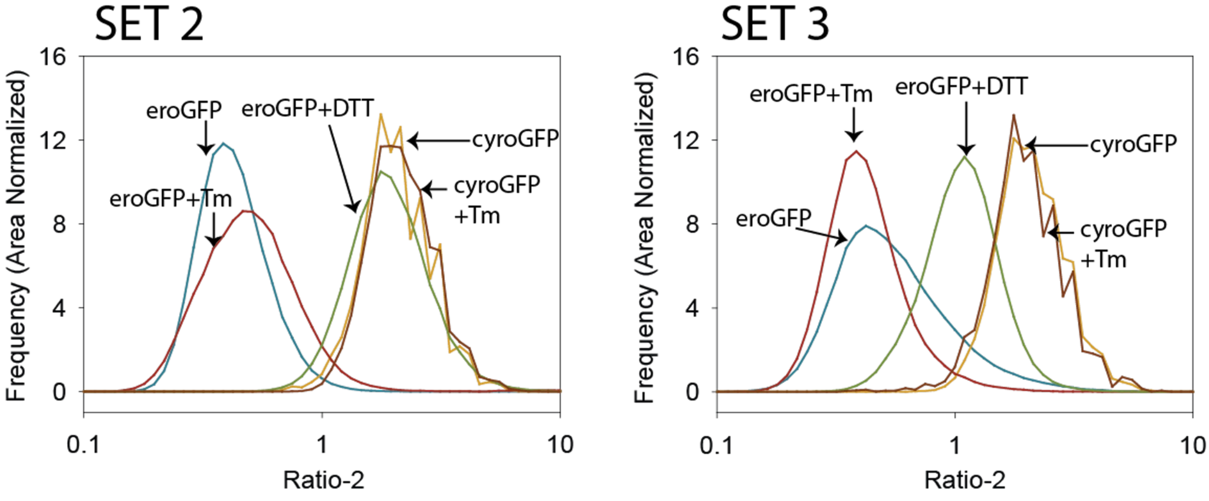


**Supplementary figure S 7: Ratio-2 distribution of primary cell culture from 1 dpf zebrafish embryos.** 1 dpf zebrafish embryos were processed to get single cell suspension followed by growing in L15 medium in chambered coverglasses. Cells kept overnight were treated with 6 µg/ml Tm (8 h) and 10 mM DTT (1-2 min) and immediately imaged sequentially to obtain emission signals (from 505-540 nm) upon excitation at 488 nm and 405 nm. Ratio-2 values obtained after image analysis were plotted separately for each condition in both Sets. Each Set and each line represents data from one replicate and mean Ratio-2 for each treatment, respectively. N is variable in each condition and contains value from at least 6 fields. Abbreviations- dpf= days post fertilization Tm= tunicamycin, DTT= Dithiothreitol.

Supplementary figure S8

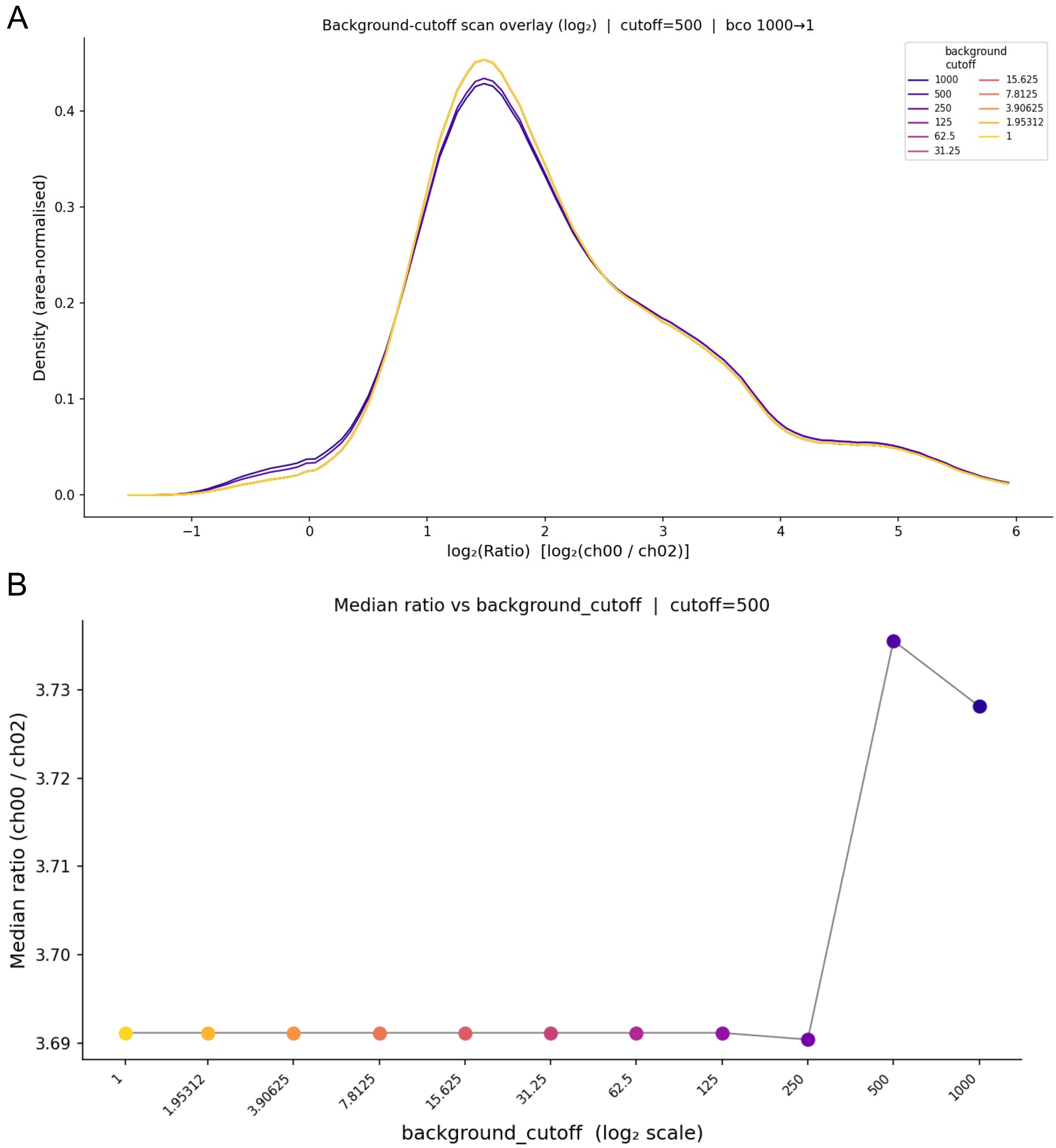


**Supplementary figure S 8: The histogram of ratios (A), or the median ratio of the frequency distribution (B) computed from the full stack of the all the images are plotted.** Background cutoff is show on x-axis (for B), 1000 indicates a relaxed cutoff where autofluorescent pixels with the intensity of 1000 or more were removed while anything below 1000 is considered, and ratio is calculated for ch00 (488 nm excitation/505-540 emission) and ch02 (405 nm excitation/440-480 nm emission). While on the left hand extreme the cutoff is stringent, at background cutoff of 1, any pixels with intensity of 1 or more were considered as autofluorescent regions and they were removed from the calculation of the ratios. As can be seen that the stringency is higher towards the left, and as stringency increases, the median changes slightly (indicating that autofluorescence contributes when the correction is relaxed). However, as the stringency increases, any cutoff below 125 does not show any change, indicating that a cutoff of 125 or less for background autofluorescence is sufficient to obtain ratios unperturbed by autofluorescence. However, to be consistent, and stringent, the background cutoff of 10 was used in all cases.

Supplementary figure S9

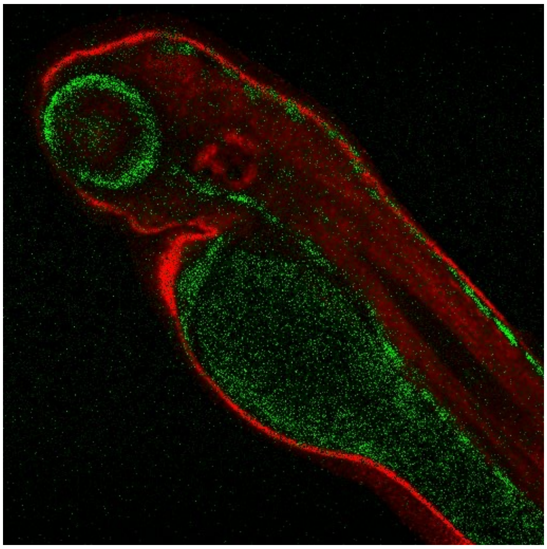

**Supplementary figure S9: A representative figure to show that the ratio is effectively calculated from the non-autofluorescent regions of the zebrafish larvae.** In red pixels show the rescaled image of ratio calculated after removing the auto-fluorescent pixels, and green pixels show the autofluorescence regions. The complimentary nature of the regions marked by red and green indicate that ratio is effectively calculated only for regions that are not auto-fluorescent, thereby reporting the true redox ratio. Image is rotated and flipped to keep it in this orientation (confocal microscope, 10 X objective).

Supplementary figure S10

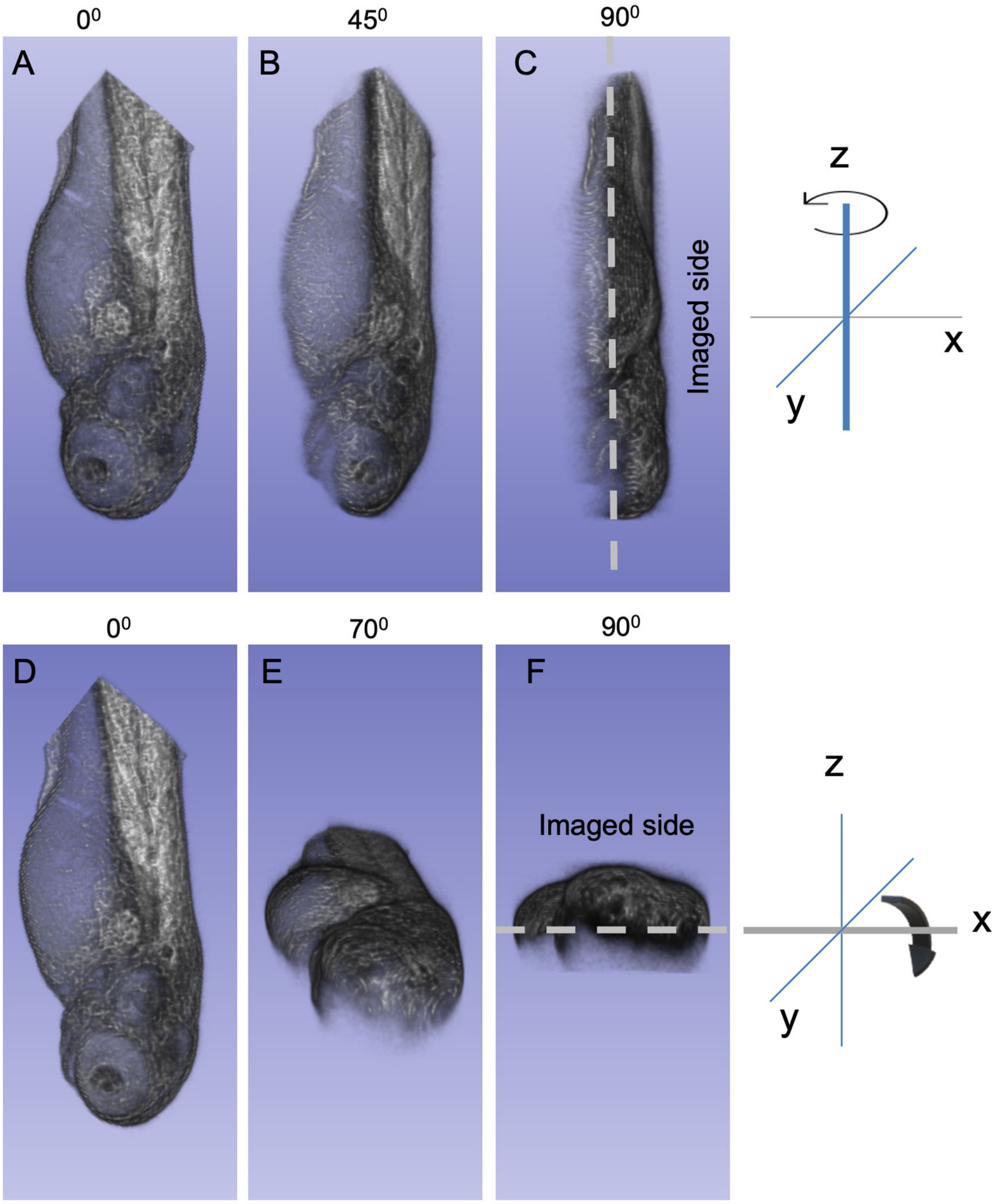


**Supplementary figure S10 : Confocal imaging enables to procure signals from either half of the embryo in 3D.** 3D representation of a 3 dpf zebrafish embryo was reconstructed from z-stack images of GFP fluorescence. A single embryo is shown for illustrating the orientations used in all the figures. (A) and (D) is the $0^0$ angle of image showing the way embryo was imaged. (B) and (C) represents the embryo at $45^0$ and $90^0$ respectively when turned left to right as indicated in the arrow. (E) and (F) represents the embryo at $70^0$ and $90^0$ respectively when turned up from down (shown in arrow) (Confocal microscope, 10X objective). Abbreviations- dpf= days post fertilization.

Supplementary figure S11

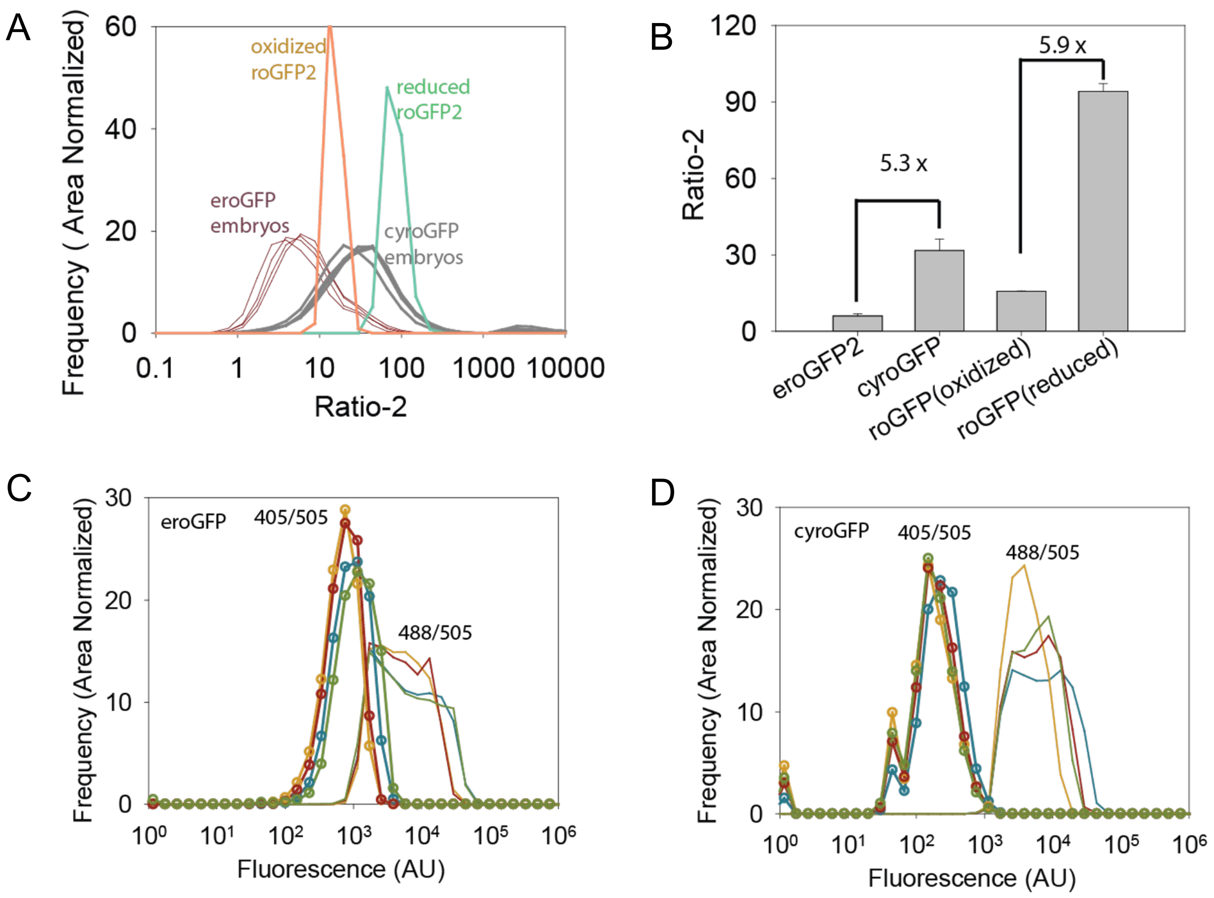


**Supplementary figure S 11: Ratio-2 distribution of eroGFP and cyroGFP fish lines showed shift in the peak as compared to the oxidized and reduced roGFP2 solution.** 3 dpf embryos were anaesthetized, mounted on soft agar, and imaged for obtaining emission signals (505-540 nm) when excited at 488 nm and 405 nm sequentially. 5 μM roGFP2 solution was treated with 10 mM DTT and 10 mM diamide to get fully oxidized and reduced species. These solutions were also imaged using similar settings. Ratio-2 values were calculated using MATLAB. (A) The frequency of each value was area normalized using the total number of pixels and graph was plotted (x axis in log10 scale). (B) Bar graph showing mean ratio-2 for the embryos as well as roGFP2 solution (oxidized and reduced) and error bars represent standard deviation. (C) and (D) Fluorescence intensities obtained with excitation at 405 nm and emission at 505 nm (405/505) and excitation at 488 nm and emission at 505 nm (488/505) were calculated for embryos. Frequency (area normalized) was plotted against fluorescence intensity for 405/505 and 488/505 for eroGFP (left panel) and cyroGFP (right panel) embryos. Each line represents one embryo in (A), (B), and (D) (number of embryos, n= 4; confocal microscope, 10X objective). Abbreviations- dpf= days post fertilization, DTT= Dithiothreitol.

Supplementary figure S12

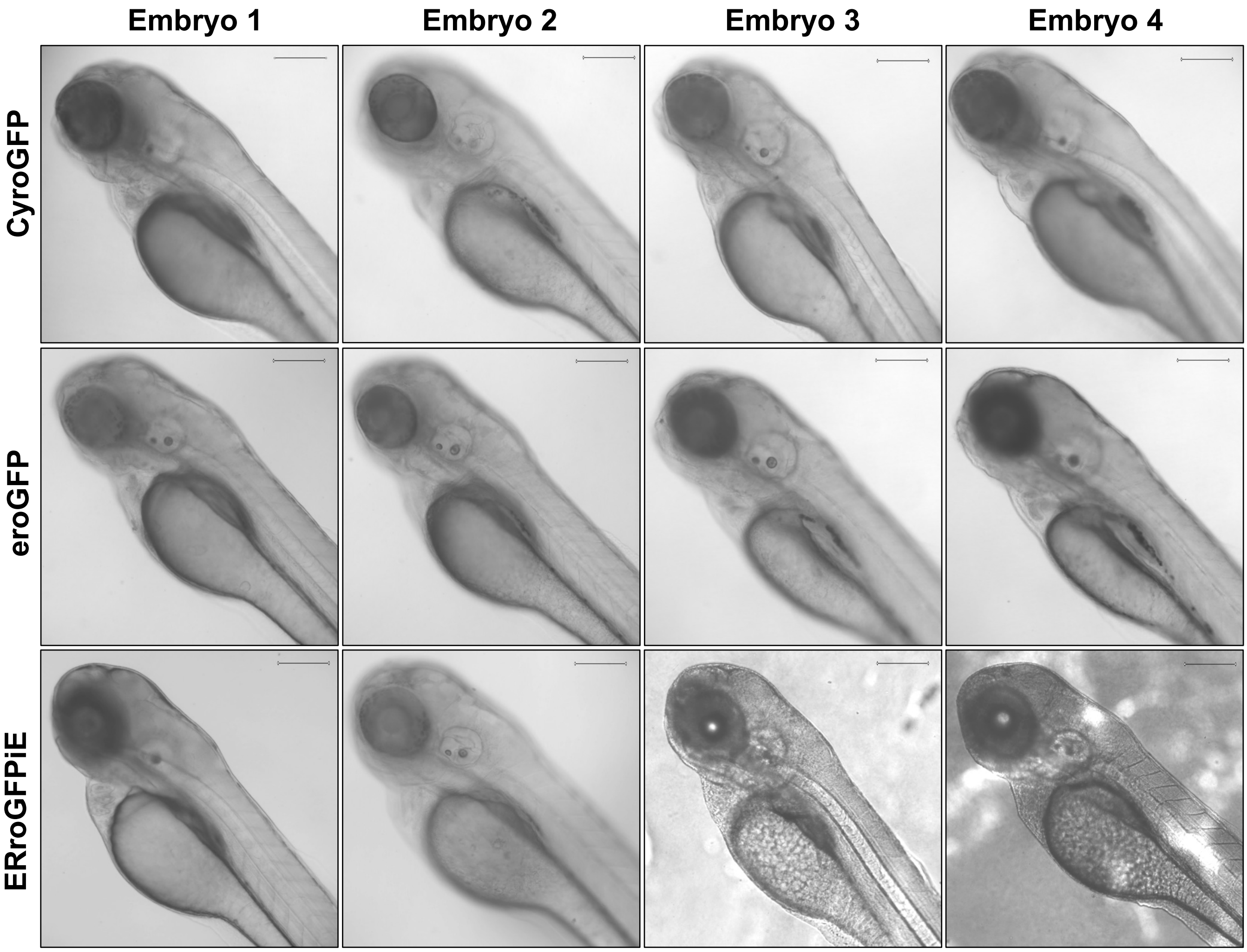


**Supplementary figure S 12: Bright field images of embryos shown for redox imaging.** Upper panel images belongs to cyroGFP line displayed in figure 5, middle panel is eroGFP embryos showed in figure 6. Lower panel had embryos showed in Figure 7. Embryo images are rotated and flipped to put them into same orientation (confocal microscope, 10 X objective, scale bar- 200 µm).

Supplementary figure S13

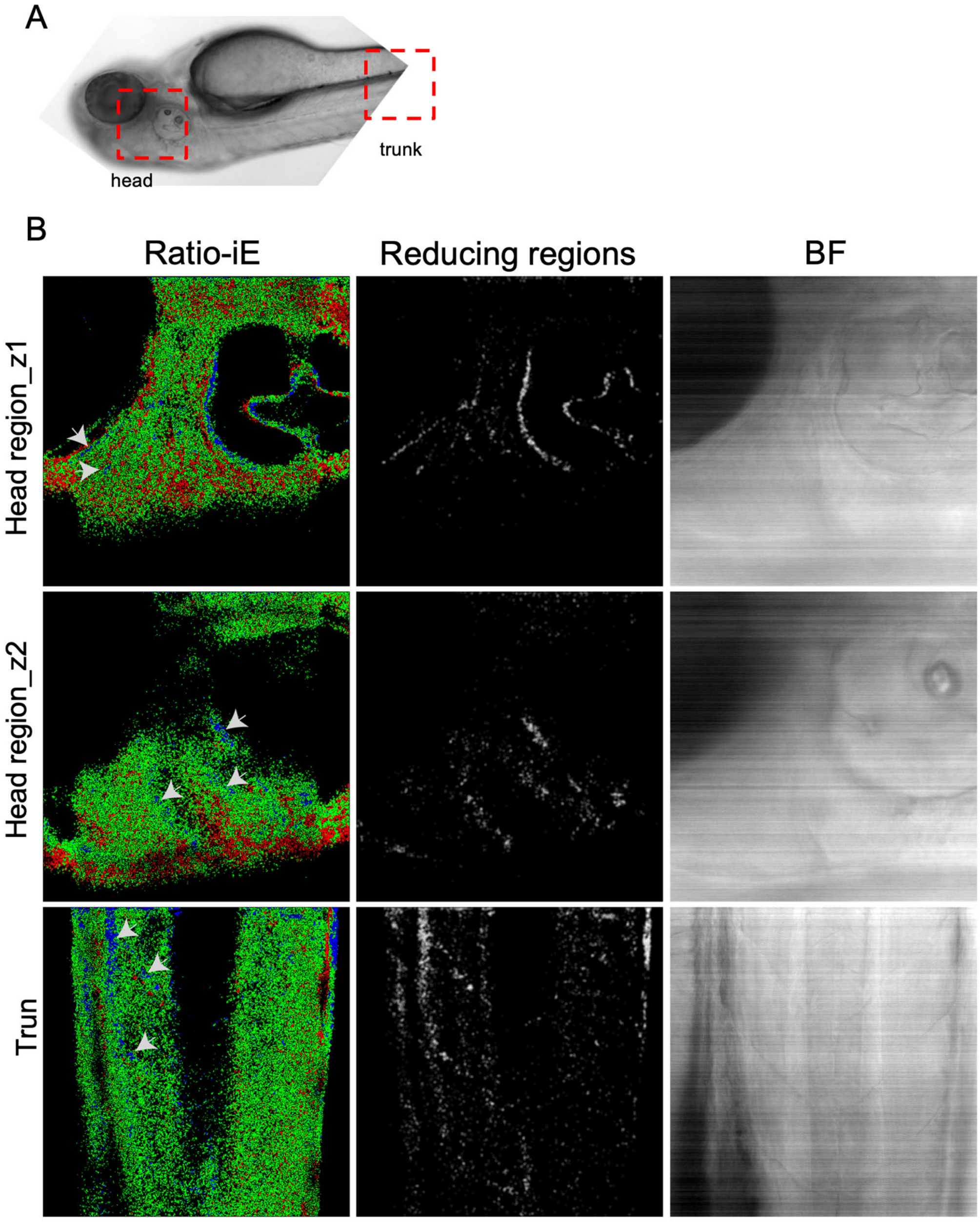


**Supplementary figure S13: Variations in redox ratios of ER are more apparent with higher magnification images of ERroGFPiE embryos.** 3 dpf live ERroGFPiE embryos were anaesthetized, mounted in soft agar, imaged, analyzed. Ratio-iE values were calculated using MATLAB and color coding was done as described by ERroGFPiE embryos. (A) A 10X BF image of an embryo showing head and trunk regions (red squares) imaged at higher magnification of 40X. (B) All three panels show representative image from one embryo taken at different regions. Left, middle, and right panel show Ratio-iE, only reducing pixels, and BF images respectively. Upper panel and middle panel shows the Ratio-iE in the eye region (black region on top left) images taken at different z depths inside the embryo. Some distinct reducing regions are marked with white arrows. Lower panel shows the Ratio-iE images taken at the trunk regions showing patches of reduced regions (white arrows) (confocal microscope, 40X objective). Abbreviations- dpf= days post fertilization, BF- bright field.

Supplementary figure S14

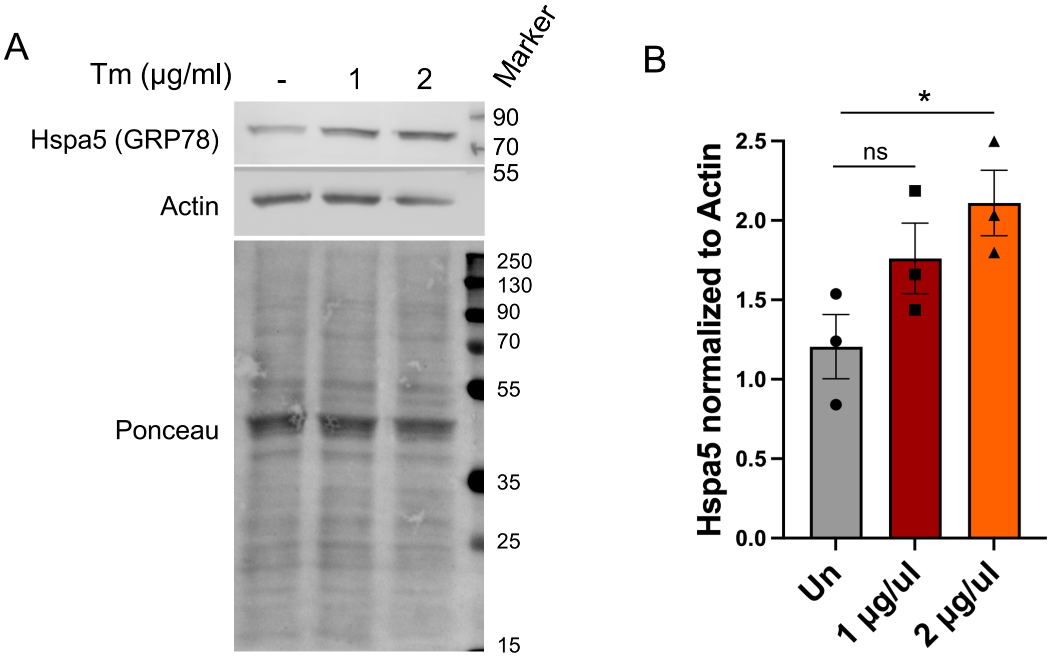


**Supplementary figure S 14: Tm treatment induced misfolding stress revealed by upregulation of Hspa5.** A and B. Approximately 40 3-dpf WT embryos were treated with Tm (1 and 2 µg/ml; n=3) for 12 h followed by harvesting and lysis. Probing was done with Hspa5 and actin antibodies. Ponceau was also done as shown in lower panel. B. Quantification of Hspa5 expression was done using ImageJ and the graph was plotted using GraphPad prism. Student's t test with unequal variance was done to test significance using Microsoft excel; * is p value <0.05 . Abbreviations- Un- untreated control, Tm= tunicamycin, dpf= days post fertilization.

Supplementary table S1. Primers used for the generation of transgenic lines

| S. no. | Primer name | Primer sequence (5'-3') | Description |
|---|---|---|---|
| 1 | zf.er.erogfp.infr1 | Ttttgctggtggccggcagcgtgtttgccat ggtgagcaagggcgaggagctgttcacc | fusing hspa5 ER SS with roGFP |
| 2 | zf.er.erogfp.fr | Agtcagaattcatgcggttgctttgcctgttttt gctggtggccggcagcgtgtttgcc | Amplification of zebrafish eroGFP forward primer |
| 3 | er.erogfp.rv | Actaagcggccgcttacaattcgtcgtgctt gtacaattcgtccataccg | Amplification of roGFP, reverse primer |
| 4 | zf.er.erogfp.NotI.fr | Agtcagcggccgcatgcggttgctttgcctg tttttgctggtggccggcagcgtgtttgcc | Amplification of zebrafish eroGFP, final forward primer |
| 5 | zf.er.erogfp.ClaI.rv | Actaaatcgatttacaattcgtcgtgcttgta caattcgtccataccg | Amplification of zebrafish eroGFP, final reverse primer |
| 6 | CyroGFP2_pSS550 _for_ZF | ccatctagagcggccatgagtaaaggaga agaacttttca | Amplification of cyroGFP2, forward primer |
| 7 | CyroGFP2_pSS550 _rev_ZF | tctggatcatcatcgttatttgtatagttcatcc atgcca | Amplification of cyroGFP2, reverse primer |
| 8 | ERroGFPiE_pSS550 _in_for1_ZF | Gacgataagaaggagagtgttgggagta aaggagaagaacttttcac | fusing hspa5 ER SS with roGFP1/iE, inner forward primer |
| 9 | ERroGFPiE_pSS550 _for2_ZF | Gtggccggcagcgtgtttgccgaagagga cgataagaaggagagtgttgg | fusing hspa5 ER SS with roGFP1/iE, 2nd forward primer |
| 10 | ERroGFPiE_pSS550 _for3_ZF | Atgcggttgctttgcctgtttttgctggtggcc ggcagcgtgtttgccga | fusing hspa5 ER SS with roGFP1/iE, 3rd forward primer |
| 11 | ERroGFPiE_pSS550 _out_for4_ZF | ccatctagagcggccatgcggttgctttgcc tg | Amplification of zebrafish roGFP2/iE, final forward primer |
| 12 | ERroGFPiE_new_R ev_pSS_ZF | Tctggatcatcatcgctacagctcgtcctttt gtatagttcatccatgc | Amplification of zebrafish roGFP2/iE, final reverse primer |
